# An archived activation tagged population of *Arabidopsis thaliana *to facilitate forward genetics approaches

**DOI:** 10.1186/1471-2229-9-101

**Published:** 2009-07-31

**Authors:** Stephen J Robinson, Lily H Tang, Brent AG Mooney, Sheldon J McKay, Wayne E Clarke, Matthew G Links, Steven Karcz, Sharon Regan, Yun-Yun Wu, Margaret Y Gruber, Dejun Cui, Min Yu, Isobel AP Parkin

**Affiliations:** 1 Agriculture and Agri-Food Canada, Saskatoon Research Centre, 107 Science Place, Saskatoon, S7N 0X2, Canada; 2 Cold Spring Harbor Laboratory, 1 Bungtown Road, Cold Spring Harbor, NY 11724, USA; 3 Department of Biology, Biosciences Complex, Queens University, Kingston, Ontario, K7L 3N6, Canada

## Abstract

**Background:**

Functional genomics tools provide researchers with the ability to apply high-throughput techniques to determine the function and interaction of a diverse range of genes. Mutagenised plant populations are one such resource that facilitate gene characterisation. They allow complex physiological responses to be correlated with the expression of single genes *in planta*, through either reverse genetics where target genes are mutagenised to assay the affect, or through forward genetics where populations of mutant lines are screened to identify those whose phenotype diverges from wild type for a particular trait. One limitation of these types of populations is the prevalence of gene redundancy within plant genomes, which can mask the affect of individual genes. Activation or enhancer populations, which not only provide knock-out but also dominant activation mutations, can facilitate the study of such genes.

**Results:**

We have developed a population of almost 50,000 activation tagged *A. thaliana *lines that have been archived as individual lines to the T_3 _generation. The population is an excellent tool for both reverse and forward genetic screens and has been used successfully to identify a number of novel mutants. Insertion site sequences have been generated and mapped for 15,507 lines to enable further application of the population, while providing a clear distribution of T-DNA insertions across the genome. The population is being screened for a number of biochemical and developmental phenotypes, provisional data identifying novel alleles and genes controlling steps in proanthocyanidin biosynthesis and trichome development is presented.

**Conclusion:**

This publicly available population provides an additional tool for plant researcher's to assist with determining gene function for the many as yet uncharacterised genes annotated within the Arabidopsis genome sequence http://aafc-aac.usask.ca/FST. The presence of enhancer elements on the inserted T-DNA molecule allows both knock-out and dominant activation phenotypes to be identified for traits of interest.

## Background

The adoption of *Arabidopsis thaliana *as a model plant was suggested as early as 1943, yet its prominence in the study of plant genetics and physiology did not emerge until the 1980's with the recognition that its small genome and ease of manipulation offered the opportunity to mutate and study every gene within the genome [1]. The ability to fully realise this objective has been facilitated through the development of an elegantly simple transformation system [2] and the completion of the genome sequence [3]. The most recent annotation of the genome sequence has identified a total of 33,282 genes comprising 27,235 protein coding genes, 4,759 pseudogenes or transposable elements and 1,288 non coding RNAs (TAIR8 release; http://www.arabidopsis.org). Computational biology tools allow the potential function of almost half of these proteins to be inferred, which provides an enormous resource for hypothesis driven research, while the remaining unknown proteins present an intriguing palette for curious researchers.

The development of tools to elucidate the function of the inferred genes is required in order to exploit the potential wealth of information provided by the annotated genome sequence. Large scale random mutagenesis has been utilised to successfully address the knowledge gap between sequence and function in a number of plant species [4-6] and has been widely applied in *A. thaliana *[7]. Numerous strategies have been employed to saturate the genome, including exposure to chemical mutagens such as ethyl methanesulphonate (EMS) [8], transposon tagging [9], fast neutron deletion [10] and agrobacterium-mediated T-DNA mutagenesis [11]. While EMS mutagenesis has the advantages of ease of application, non-biased distribution across the genome and generation of subtle phenotypes, its utility has been somewhat limited by the time-consuming map-based cloning required to verify the underlying gene responsible. The use of specific DNA insertional elements, such as transposons and T-DNAs, allows the rapid identification of the point of entry in the genome using PCR based protocols, which have been optimised for high throughput sequencing [11,12]. The generation of large collections of mutagenised lines and the concurrent sequencing of insertion sites to develop readily searchable databases for these populations has revolutionised gene characterisation by providing 'in silico' access to thousands of mutant alleles.

The Arabidopsis community is fortunate that a number of populations are readily available for reverse genetics applications and can be accessed through The Arabidopsis Information Resource (TAIR: http://www.arabidopsis.org). In total, three publicly available T-DNA flanking-sequence tag (FST) databases provide access to over 200,000 insertion sites; SIGnAL, FLAGdb and GABI-Kat [11,13,14], which have been estimated to interrupt the transcription of 80% of the annotated protein coding genes [15].

Although the utility of T-DNA mutagenesis has been enhanced through the use of vectors that can facilitate gene, enhancer or promoter trapping [16], there is an inherent limitation to simple insertional mutagenesis due to functional redundancy within the genome. Approximately 17% of *A. thaliana *genes are found in direct tandem repeats and 58% of the genome is thought to be duplicated, providing the plant with the ability to compensate for many null mutations [3]. The development of vectors which can generate gain-of-function as well as loss-of-function alleles, so called activation tagging, has led to the discovery of a number of novel alleles controlling important functions in plant development, metabolism and stress responses [17]. Activation tagging exploits a tetrameric repeat of the enhancer element of the cauliflower mosaic virus (CaMV) *35S *gene to direct the transcription of adjacent genes generating dominant phenotypes [18]. Although a number of resources have been developed for *A. thaliana *using this strategy [18,19], access to these lines is generally via pooled seed samples or through databases of predetermined visual phenotypes (http://www.arabidopsis.org; http://amber.gsc.riken.jp/act/). In addition, Ulker et al (2008) [20] recently observed unanticipated activation and anomalous expression events in what would traditionally be considered knock-out populations suggesting that such populations may harbour novel phenotypes.

This study describes the generation of an archived activation tagged T-DNA *A. thaliana *(ecotype Columbia) population derived from almost 50,000 individual T_1 _lines, where to date at least 19,000 flanking sequence tags (FSTs) have been identified to facilitate reverse and forward genetics applications http://aafc-aac.usask.ca/FST. The distribution of the integration events in the genome was investigated and found to be closely correlated with gene density and not with recombination frequency although a reduction in frequency was observed across all datasets in centromeric regions. The analyses identified the presence of novel alleles, multiple insertions sites, complex Ti plasmid integrations and the somewhat unexpected assimilation of agrobacterium sequences into the genome. The utility of the described population for identifying new mutations controlling a number of physiological traits is being explored and preliminary phenotypes are presented for trichome development and proanthocyanidin metabolism.

## Results

### Generation of the SK Population

An *A. thaliana *T-DNA mutagenised population, named SK, was developed and archived as T_2 _seed derived from 49,160 individual herbicide resistant T_1 _lines with a T-DNA transformation efficiency estimated to be ~0.05%. Single seed descent with continued selection was employed to generate a population of 44,383 T_3 _families that will be enriched for homozygous mutant genotypes.

The number of independent insertion events per line was estimated initially by assessing the segregation ratio for herbicide resistance scored in the progeny from 100 T_1 _plants. This resulted in an estimate of 1.35 insertion loci/line suggesting the entire population may contain ~70,000 independent T-DNA integration events. However, Southern analysis of 102 lines suggested a greater number of actual integration events (3.1 T-DNA insertions/line) with a high percentage (~82%) of the insertion alleles being the result of complex T-DNA integrations events (data not shown). This was later confirmed through sequence analysis of the DNA flanking the T-DNA left border (see below), which is in contrast with the lower frequency of T-DNA integration reported in previously characterised populations [11,21].

### Genomic Distribution of Flanking Sequence Tags (FSTs)

TAIL-PCR was employed as a relatively efficient high-throughput strategy to amplify the sequence flanking the T-DNA insertion events (FST) present in the SK mutagenised population [12]. The genetic origin of 16,428 FST sequences derived from DNA flanking the left border of stably inherited T-DNA molecules was determined by analysing the sequence from amplification products generated from 28,908 individual T_2_lines. Additional sequencing is on-going to characterise further SK lines.

The genomic location of the integrated T-DNA molecules was determined by aligning each FST sequence with the five nuclear and two extra-nuclear *A. thaliana *pseudochromosomes. The T-DNA integration sites were classified based on the available annotation (TAIR8; http://www.arabidopsis.org) and the frequency of integration in promoter, 5'-UTR, exon, intron, 3'-UTR and intergenic regions was determined (Table 1). This initial survey revealed integration events in 8,324 (25% of the annotated *A. thaliana *genes) unique gene regions including promoter sequence, with 36% of these insertion events predicted to interrupt exons. T-DNA integration events were observed more frequently in the untranslated sequences (5'UTR χ^2 ^= 1,035, p < 0.0001; 3'UTR χ^2 ^= 545, p < 0.0001) and less frequently in intron and exon sequences (χ^2 ^= 941, p < 0.0001; χ^2 ^= 719, p < 0.0001) than expected based on their relative proportion of the annotated genome.

**Table 1:** Position and number of SK FST Integrations in the *A. thaliana *genome.

		Chr1	Chr2	Chr3	Chr4	Chr5	eChr^a^	Total
Promoter	Hits^b^ Genes^c^	837 640	583 413	676 514	468 361	871 629	0 0	3,435 2,557

5'-UTR	Hits Genes	273 233	126 101	200 161	157 129	217 192	0 0	973 816

Exon	Hits Genes	883 733	763 535	756 609	626 484	811 637	0 0	3,839 2,998

Intron	Hits Genes	455 374	298 248	345 288	285 231	411 336	0 0	1,794 1,477

3'-UTR	Hits Genes	296 255	180 154	245 207	174 153	283 237	0 0	1,178 1,006

Intergenic	Hits Genes	1,410 n/a	817 n/a	1,002 n/a	835 n/a	1,139 n/a	6 n/a	5,209 n/a

Total	Hits Genes	4,154 2,118	2,767 1,338	3,224 1,671	2,545 1,281	3,732 1,916	6 0	16,428 8,324^d^

a. eChr represents the two extra-nuclear genomes.

b. Number of independent T-DNA integrations.

c. Number of independent disrupted genes.

d. Number of unique genes with T-DNA insertions.

**Figure 1:**
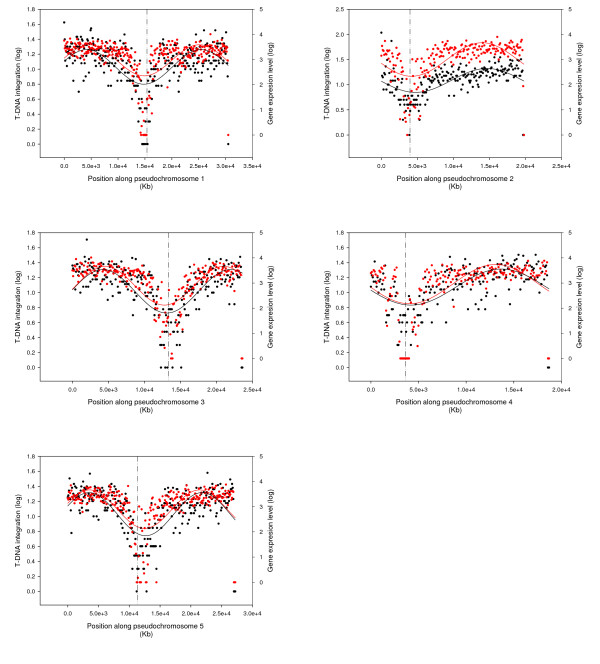
**Distribution of T-DNA integrations along each *A. thaliana *chromosome**. The number of T-DNA integrations (black) and the level of gene expression (red) in each 100 Kb window along the chromosome was determined (log_10 _scale shown). The curved and dashed lines represent the line of best fit for each distribution and the position of the centromere, respectively.

The distribution of T-DNA integration sites was not uniform, with many regions of the genome possessing either an over abundance or a dearth of insertion events (Figure 1; Additional file 1). The density of T-DNA insertions was compared to both the level of gene expression in carpel tissue and the rate of genetic recombination previously observed for *A. thaliana *[22]. There was strong correlation between the level of gene expression and the frequency of T-DNA integration, but no correlation with recombination frequency along each chromosome; although a stark reduction in gene expression, recombination and T-DNA insertion frequency was observed in the centromeric regions (Figure 1).

### Nearing a mutation saturated *Arabidopsis thaliana* genome

**Figure 2:**
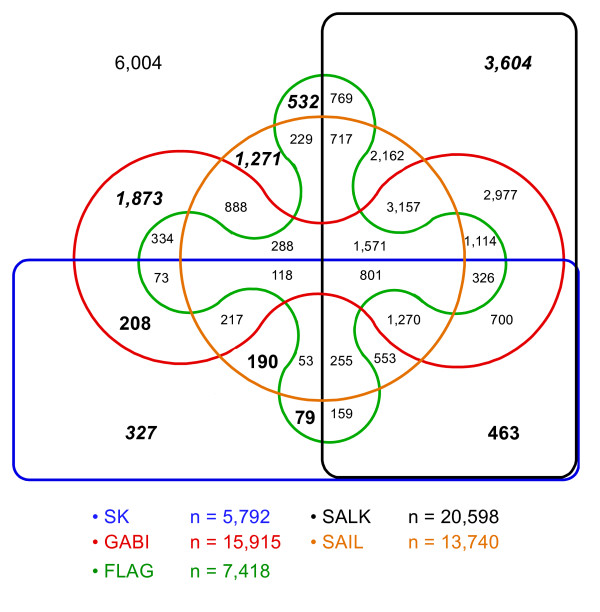
**Edwards Venn diagram showing the overlap among genes harbouring a T-DNA insertion within five *A. thaliana *FST populations**. The number of loci with an insertion in a single population is shown in bold italic font. The number of loci where a second allele is found in the SK population is shown in bold font.

The SK FST data combined with available sequence data from previously established T-DNA mutagenised populations of *A. thaliana*, SIGnAL [11], FLAGdb [13], SAIL [12] and GABI-Kat [14], revealed that the Arabidopsis genome is reaching complete saturation with knock-out alleles now available for 27,324 (82%) of the annotated genes (Table 2). When considering only those FSTs residing in exon sequences, which are the mutations most likely to generate loss of function alleles, this number was reduced to 23,556 and represented 71% of the annotated genes (Table 2). By assessing all populations, 20,296 (61%) genes with multiple independent potentially deleterious alleles were identified, of which 13,119 (40%) genes possessed multiple alleles with interrupted exon sequences. Unique insertion events have been identified in each population in proportion to the depth of FST sequence capture (Figure 2). In particular, the SK population provides 327 novel insertion events in *A. thaliana *genes and a second allele for 940 genes.

**Table 2:** Summary of the publicly available *A. thaliana *T-DNA insertion events.

*T-DNA population*	*Ecotype*^a^	*FST-capture method*	*No. of FST's*^b^	*FSTs in genes including promoters*	*FSTs in transcribed regions*	*FSTs in exons*
SK	Col	TAIL-PCR	16,428	11,219 ^c ^(8,324)^d^	7,813 (5,792)	3,758 (2,981)

SALK	Col	Genome walking	145,589	93,945 (24,589)	70,348 (20,598)	37,513 (15,139)

SAIL	Col	TAIL-PCR	57,242	42,788 (17, 230)	35,158 (13,740)	20,116 (9,006)

GABI	Col	Genome walking	63,887	41,624 (19,989)	31,251 (15,915)	16,684 (10,618)

FLAG	Ws	Genome walking	31,744	17,863 (10,798)	11,800 (7,418)	5,155 (3,755)

**Total FSTs**			**314,886**	**207,439 (29,321)**	**156,370 (27,324)**	**83,226 (23,556)^e^**

*a. A. thaliana *ecotypes: Col – Colombia; and Ws – Wassilewskija.

b. Number of FSTs assigned to a unique position within the *A. thaliana *genome.

c. Number of recorded FSTs.

d. Number of unique genes interrupted by an FST.

e. Total number of unique genes with an insertion.

### Characterisation of the *A. thaliana* genes without insertions

There remain 6,004 *A. thaliana *genes with no identified T-DNA insertion event when all available populations are considered. After removing 1,550 annotated gene codes that were less than 200 bp in length (largely consisting of tRNAs, microRNAs, and retrotransposons), a number of basic characteristics were assessed for each of the remaining genes. These included gene expression level from carpel tissue, position relative to the centromere, annotated length, and gene copy number (Additional file 2).

A significant bias in gene length was observed with the median length for genes with and without an insert being 2,418 bp and 1,132 bp, respectively (z <-100, p < 0.0001). The distributions of gene expression levels for genes with and without insertions were also distinct (z = -21.99, p < 0.0001). The median absolute expression level was seven-fold lower for those genes without an insertion compared to those having a T-DNA integration event. This observation correlated with the position of the genes relative to the centromere, where gene expression is repressed, since those genes lacking an insertion event were found to be demonstrably closer to the centromeric region (z = -30.76, p < 0.0001). Similarly, pseudogenes that are generally not expressed or expressed at low levels were three-fold over-represented among the gene annotations for gene codes with no observed T-DNA integration.

### Identification of complex T-DNA and non-Ti plasmid integration

**Figure 3:**
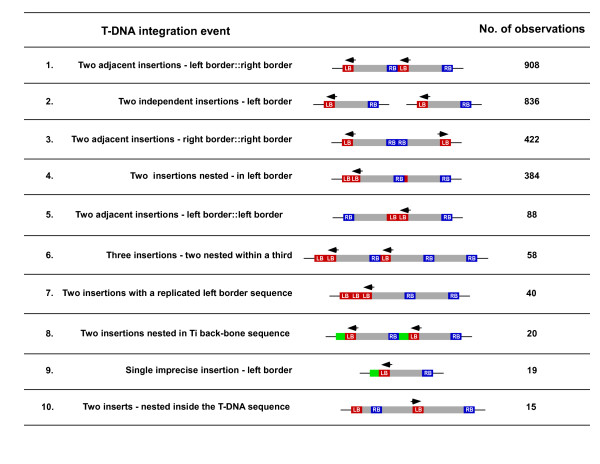
**Types and frequency of complex T-DNA insertion events within the SK population**. Complex T-DNA integration events fell into ten classes, differentiated by the number of times a border sequence was present, the presence of Ti plasmid or internal T-DNA sequence and the strand orientation. Red and blue boxes indicate the left and right border sequences, respectively. Green boxes represent pSKI015 backbone sequence, and the arrowhead shows the priming site that generated the observed FST sequence.

Based on visual analysis of the FST sequence chromatogram files it was apparent that some of the FST sequences represented multiple amplification products (data not shown). Further analyses of the FST database identified 836 SK lines harbouring two independent T-DNA integration events (Figure 3, No. 2) and an additional 1,954 lines (10%) with complex T-DNA integration events (Figure 3, Additional file 3). Figure 3 depicts the type and frequency of each complex insertion event observed, 73% of which were back-to-back tandem insertion events, with the majority being found in the left border-right border (LB: RB) orientation. A portion (25%) of the remaining lines contained a second left border sequence or internal T-DNA vector sequence which identified a nested integration event. In a small percentage of lines imprecise transfer of the T-DNA resulted in integration of Ti vector backbone sequence adjacent to the left border. An additional 35 SK lines contained segments of *Agrobacterium tumefaciens *genomic sequence, the majority of which (32 lines) originated from the linear chromosome of *A. tumefaciens*. This phenomenon was recently observed by Ulker et al (2008) [23] and suggests that transfer of bacterial genomic DNA occurs at a low but discernable rate during Agrobacterium plant transformation.

### SK FST data handling and visualisation

**Figure 4:**
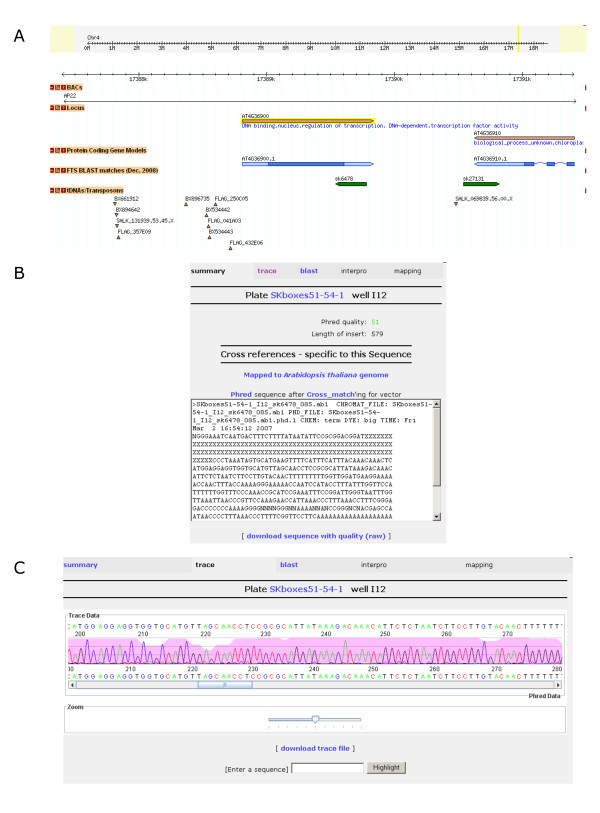
**Web interface for the display of FST sequence features in the context of the *A. thaliana *genome http://aafc-aac.usask.ca/fst/**. A 5 kb view around a T-DNA insertion harboured by the SK6478 line is shown. FST sequences are visualized using a standard GBrowse genome viewer (A). Users may obtain detailed sequence information (B) from our sequence portal including sequence traces (C).

The DNA sequencing data for each SK line was warehoused using APED (http://sourceforge.net/projects/aped Figure 4b). Each FST was aligned to the genome sequence of *A. thaliana *and the resulting sequence similarity was used to represent the insertion site locations within Gbrowse [24] (Figure 4a). The DNA sequencing data (Figure 4c) as well as the visualization relative to the *A. thaliana *genome are available http://aafc-aac.usask.ca/FST.

### Forward Genetic Screens reveal novel mutations

Aberrant morphological variation was observed in individual lines throughout the generation of the SK population and a number of these were confirmed as alleles of previously characterised mutations through the mapping of the FSTs. Some examples of these included mutations in *APETALA1 *(At1g69120; SK295), *LEAFY *(At5g61850; SK14914), and *CABBAGE *(At5g05690; SK4745). In addition to loss-of-function alleles, gain-of-function mutants should also be discovered since the SK population was developed using a vector carrying multiple enhancer elements. Activation of genes adjacent to the insertion site was confirmed for at least two phenotypic variants, one leading to ectopic expression of a gibberellin oxidase resulting in a dwarf phenotype [25] and the second to activation of an adjacent microRNA resulting in enhanced seed carotenoid levels (Wei et al, submitted).

To fully realise the potential of this genetic resource, a number of forward genetic screens were initiated to identify lesions in targeted developmental and biochemical pathways. The preliminary results from two screens dissecting trichome development and proanthocyanidin accumulation in the seed coat are presented.

**Figure 5:**
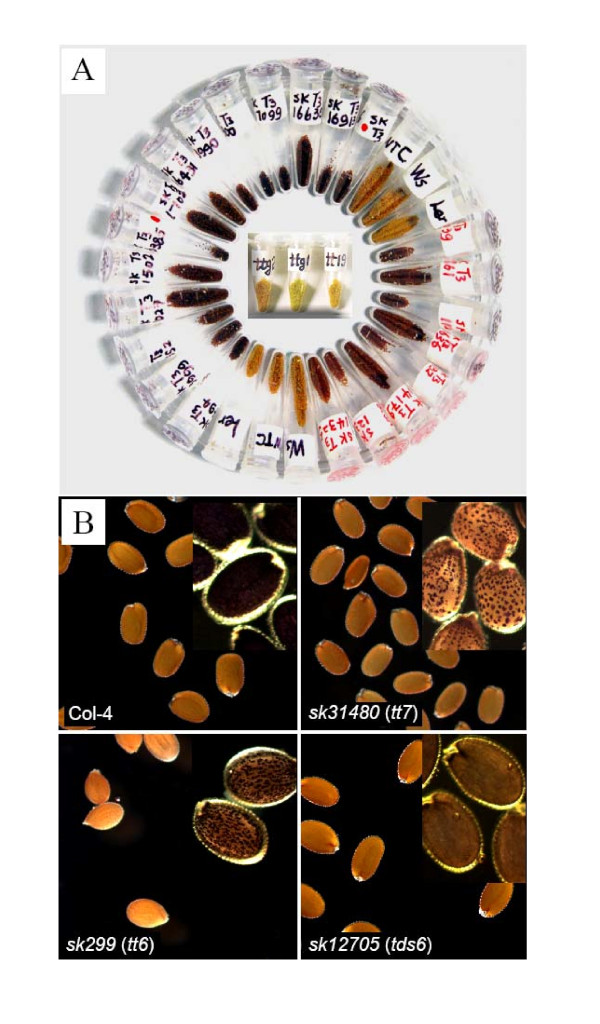
**Seed coat colour and proanthocyanadin depositions represented in the SK population**. A) Variation in seed coat colour of selected SK mutant lines compared to wild type ecotypes Columbia (WTC), Wassilewskija (WS) and Landsberg (Ler) that are medium brown in colour and known *transparent testa *(*tt*) mutants (centre of image). B) Large panels show visible seed colour patterns. Small inserts show close ups of dark, DMACA-stained, streaked proanthocyanidin patterns in Col-4 and spotted or patchy patterns in two mutants. A third tan coloured mutant has even colouration overlaid with tan streaks.

Fifty-one lines were selected by screening 49,160 T_3 _SK seed lines and 220 SK T_2 _seed pools for seed colour variation and proanthocyanidin patterning. Concomitant screening of 20,200 T_2 _non-activation T-DNA lines (those containing no 35S enhancer sequences) did not realise any seed colour variants. Based on visual inspection in comparison to wild type, selected lines were divided into colour categories, ranging from dark brown to yellow (Figure 5A). The seed coat phenotype for most of these lines appeared similar to published *transparent testa *(*tt*) or *tannin deficient seed *(*tds*) mutants after histochemical staining (Figure 5B). Further studies have revealed altered phenotypes (named *sk-tt *mutations) resulting from mutant alleles of seven genes already known to be involved in proanthocyanidin biosynthesis. In addition, on-going analysis of four proanthocyanidin variants suggests their novel phenotypes are conferred by mutations affecting previously uncharacterised genes, based on diallelic crossing with known mutants and molecular characterization of the insertion sites (data not shown).

**Figure 6:**
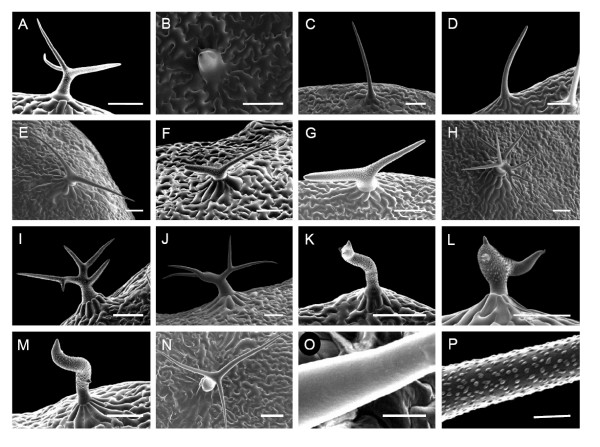
**Scanning electron micrographs of trichomes with alterations in branch number or shape**. A) wildtype, B) SK41546, C) SK270, D) SK5775, E) SK2298, F) SK4201, G) SK43953, H) SK1967, I) SK3023, J) SK42715, K) SK1824, L) SK3344, M) SK44335, N) SK8517, O) higher magnification of SK8517, P) higher magnification of wildtype. Scale bar = 100 μm for A-N, and 10 μm for O, P.

A typical wild-type *A. thaliana *leaf will have on average 97% of the trichomes with 3 branches (Figure 6A), 1% two-branched, and 2% with four-branched trichomes as based on our analysis of 798 plants. An initial set of 14,201 T_3 _SK lines were screened for alterations in trichome morphology, from which thirteen showed variation in cell shape, branch number, or the texture of the cell surface (Figure 6). SK41546 produced small trichomes of which approximately 80% lacked aerial extension of the cell similar to *glabrous *mutants, while the remaining trichomes produced partially or fully extended spikes (Figure 6B) [26-28]. SK270 (Figure 6C) and SK5775 (Figure 6D) developed branchless trichomes, 100% branchless in the SK270; however, the phenotype of SK5775 showed incomplete penetrance, such that 2–5% of the trichomes maintained two branches. In three lines, all observed trichomes displayed short stalks with two branches. In SK2298 the two branches were of similar thickness; however, in SK4201 and SK43953, one branch was thicker than the other and resembled a thumb and forefinger (Figure 6F, 6G). Three lines had supernumerary branching phenotypes similar to *kaktus *[29]. In two of these lines, SK1967 and SK3023, all trichomes showed supernumerary branches (Figure 6H and 6I), while in SK42715 at least 90% of the trichomes had 4–5 branches and the remaining appeared wild type (Figure 6J). Three lines were also identified with distorted trichome phenotypes (SK1824, SK3344, SK44335; Figure 6K, 6L, 6M) similar to the deformed trichomes of *crooked *and *distorted2 *[30,31]. The final mutant, SK8517, had normal branching, but its mature trichome lacked papillae normally present on the cell surface (Figure 6N and 6O) and were similar to the *trichome birefringence *mutant [32]. FST sequences were available for four of the thirteen trichome mutant lines, which confirmed that SK270 and SK2298 possessed alleles of *STICHEL *[33] and *ZWICHEL *[34] respectively, as suggested by their observed trichome morphology. The other two T-DNA insertions were not located near any known trichome genes.

## Discussion

Functional genomics tools are used to elucidate the role each gene plays within an organism. Due to its comparatively small size and the breadth of resources available, *A. thaliana *was a prime target to attempt a holistic assault on the genome (Arabidopsis 2010 Program: http://www.arabidopsis.org/portals/masc/FG_projects.jsp). The Arabidopsis community and indeed related species such as the important crop Brassica species have benefited greatly from the ambitious goal of assigning function to each of the ~30,000 annotated Arabidopsis genes. A number of T-DNA mutagenised populations of *A. thaliana *have been developed and released into the public domain [11-13,35,36], which greatly facilitate reverse genetic analysis of target genes through the identification of knock-out alleles.

The SK population of almost 50,000 activation tagged *A. thaliana *lines was generated and archived as T_3 _seed through single seed descent to provide a resource for forward and reverse genetic screens. The activity of the enhancer element present within the integrated T-DNA was expected to produce novel alleles and to increase the likelihood of affecting phenotypes for genes previously masked through the inherent redundancy in the *A. thaliana *genome. The SK lines carried an average of 1.35 independently segregating insertions per line. Sequencing of DNA flanking insertion sites has genetically characterised 16,428 T-DNA integration events in 15,507 SK lines. The distribution of insertion sites closely mirrored the gene content and gene expression level observed along the *A. thaliana *chromosomes, with a dearth of insertions in centromeric regions.

A comparison with previously characterised populations determined that the SK population provides 327 unique insertion events in previously untagged *A. thaliana *genes. Including the SK lines, the available populations provide multiple mutagenic alleles for 27,324 loci. Since the background mutation rate in such populations has been estimated to be as high as 60% [21] the availability of independent alleles for each gene is essential to confirm functional assignment.

Mutagenic saturation of the *A. thaliana *gene complement has yet to be achieved, since 6,004 loci still do not have a characterised T-DNA insertion event. An assessment of the loci without insertion events supports the previous analysis which suggested that T-DNA integration preferentially targets transcriptionally active regions of the genome [15]. Among the genes lacking an insertion event there was a bias towards short loci that lacked introns and were expressed at very low levels in carpel tissue (Additional file 2). This bias could explain the prevalence of transcription factors which were found among the non-mutagenised loci. Single copy genes were not over-represented among the untagged loci, which might have been expected for essential non-redundant loci. However, it is possible that such loci are being maintained within the populations in the hemizygous state.

The apparent necessity for accessible or open chromatin regions for T-DNA integration is in conflict with the observed bias of insertion events to intergenic genomic sequence compared to annotated genic regions (χ^2 ^= 1,457, p < 0.0001). There is increasing evidence that there are additional unannotated *A. thaliana *loci present in the genome [37,38] that could explain the apparent 'intergenic' insertion events. However, only 275 of the 5,209 intergenic insertion events within the SK population were associated with either the recently described 7,160 sORFs predicted from whole genome expression TILING arrays or 2,263 newly annotated proteins determined from extensive peptide sequencing [37,38]. The observed discrepancy could be accounted for by insufficient annotation of distal regulatory regions, which have been erroneously classified as intergenic sequence.

Based on the resolvable FST data, a notable number of the T-DNA integration events were found to be complex in nature (11%), predominantly indicating inverted or direct tandem insertion events. Although this implies that single genetic loci are affected, such loci complicate downstream cloning efforts and can potentially lead to additional chromosomal rearrangements [39-41].

In recent years, collections of Arabidopsis mutants (*tds *and *tt *lines) have been identified by screening for alterations in seed coat colour, flavonoid biosynthesis and proanthocyanidin accumulation [42-45]. These lines have been used to investigate the flavonoid and proanthocyanidin pathways (reviewed in [46,47]), yet the biochemical characterization of the latter stages of the pathway has been inadequate and the relative functional position of some proteins remains obscure [48-51]. The poorly characterised steps in flavonoid synthesis could be elucidated further through exploitation of the SK lines. Similarly, questions remaining on the development and regulation of trichome formation [52] could also be addressed using the described genetic resource.

The SK population is the first *A. thaliana *activation tagged population to be screened for seed coat colour, proanthocyanidin patterning, and trichome variation. To date, we have recovered a broad assortment of mutants including 12 *sk *trichome variants, seven *sk-tt *lines defining new alleles of proanthocyanidin genes and four *sk-tt *lines with as yet uncharacterised proanthocyanidin phenotypes and genes (Cui et al., Li et al., Gao et al., manuscripts in preparation). These mutant lines will add a wealth of information to our understanding of the flavonoid pathway and trichome development. The SK population already has proven its value by yielding new phenotypes and genes previously unknown to be involved in proanthocyanidin biosynthesis and regulation (Gao et al, manuscript in preparation). The population should be an excellent resource for exploring additional key processes controlling a multitude of traits and is currently being screened for mutants affected in abiotic stress tolerance and caretonoid biosynthesis.

## Conclusion

An additional resource of almost 50,000 T-DNA tagged *A. thaliana *lines has been developed enabling the continuing efforts to assign function to the entire gene complement of a plant http://aafc-aac.usask.ca/FST. This population can be screened for both loss and gain-of-function phenotypes due to the presence of enhancers on the integrated T-DNA molecule. Mapping of the FSTs for 15,507 SK lines has identified insertion events in 327 genes with no previously recorded T-DNA mutation and a second allele for 940 additional genes. The utility of this population for capturing novel phenotypic variation has been demonstrated through initial screens assaying trichome development and proanthocyanidin accumulation. The potential of this resource to genetically dissect complex physiological responses is being exploited in the study of the plant's response to abiotic stress.

## Methods

### T-DNA mediated mutagenesis

All plants were grown in a 50:50 combination of coconut fibre and soil-less mix containing slow release fertilizer under greenhouse conditions at 20°C with 16 hours light. The wild-type *A. thaliana *accession Columbia-4 (Col-4) was transformed with the binary vector pSKI015 [18] using the *A. tumefaciens *strain GV3101 according to the floral dip protocol described in Clough et al [53] with the addition of a vacuum infiltration step subsequent to submersion of the plants in the GV301 culture, when a vacuum of 500 mm/Hg was applied and held for 3 minutes (Gast model DOA-P104-AA vacuum pump, Fisher Scientific, Ottawa, Canada). Primary transformants (T_1_) were selected using the herbicide glufosinate ammonium (Liberty, Syngenta, Canada). The primary transformants were transplanted into Arasystem trays (BetaTech, Belgium) to allow maintenance of individual lines. In total, 49,160 T_1 _activation tagged lines (SK lines) were generated from which T_2 _seed was archived and T_3 _lines were obtained by single-seed descent. Plant tissue (50–100 mg) was collected from each T_2 _line into 96-well microtube racks (Qiagen Inc., USA) and stored at -80 C. Samples were freeze-dried, homogenized in a Retsch MM300 mill and DNA was extracted using a CTAB method based on Doyle and Doyle (1990).

### Estimation of insertion site number

The segregation ratio of herbicide resistance seedlings were obtained for 100 T_2 _lines by growing ~50 seeds on Petri plates using the methods described in Robinson et al., (2004) with the addition of 7.5 mg/litre glufosinate ammonium (Bayer CropScience, Regina, SK). The segregation ratio for herbicide resistance was scored 14 days after plating, which was assumed to reflect the number of independent insertions.

### Amplification and sequencing of flanking sequence tags (FST)

The genomic sequence flanking the T-DNA insertion sites (FST) was amplified by mTAIL-PCR as described in [12] using the T-DNA specific primers pSKTAIL-L1: TTCTCATCTAAGCCCCCATTTGG and pSKTAIL-L2: TGGACGTGAATGTAGACACGTCG. The FST sequence was generated using primer pSKTAIL-L3: ATACGACGGATCGTAATTTGTCG. DNA sequence was obtained using 2 μl of the purified product and Big Dye v3.0 chemistry in accordance with the manufacturer's instructions and resolved on an Applied Biosystem 3700 sequencer (Applied Biosystems, Foster City, US).

### Sequence Analysis

Custom Perl scripts were developed to delimit the boundaries and orientation of each gene feature (exon, intron, promoter and untranslated regions sequence) from each annotated gene in the TAIR8 release http://www.arabidopsis.org, and the proportion of the genome for each of the summed values for these gene features was determined: exon (35.9%); intron (22.2%); promoter (24.5%); 5' UTR (2.2%); 3' UTR (3.8%); and intergenic sequence (11.4%). For each SK-FST line the DNA sequencing trace files were warehoused in APED http://sourceforge.net/projects/aped. All FST sequences obtained were aligned to the five nuclear and two organellar pseudochromosomes of *A. thaliana *using the BLAST algorithm [54] with default BLAST parameters except that low-complexity filtering was disabled. These sequences have been submitted to GenBank (Accession Nos. FI978382 – FI994028). FSTs with no homology to the Arabidopsis genome were aligned to the pSKI015 T-DNA vector (AF187951) and the circular, linear and AT plasmid genome sequences of *A. tumefaciens *(AE007869; AE007870; AE007872). FSTs with ambiguous genome assignments and those containing complex repetitive elements were excluded from further analysis.

The pseudochromosome coordinate for each FST in the five Arabidopsis FST datasets (SK, SALK, GABI, SAIL, FLAG) was used to identify multiple alleles for each Arabidopsis gene identifier (AGI). Where multiple FSTs from the same dataset were assigned to a single AGI, those FSTs separated by at least 500 bp were considered to be distinct loci.

### Distribution of T-DNA insertion events

The number of FSTs in windows of 100 Kb along each pseudochromosome was determined. Expression levels for each gene was calculated as the mean signal value from three replicate measures from available Affymetrix data from carpel tissue obtained from stage 12 floral tissue as described for slides ATGE_37_A-C at http://affymetrix.arabidopsis.info/narrays/experimentpage.pl?experimentid=152. Only those genes present in three replicate samples were included in the analysis. These data were selected since the ovules are considered the target for heritable T-DNA integration events [55]. The recombination frequencies between 676 markers used to develop high resolution *A. thaliana *linkage groups and their position on each pseudochromosome was obtained from Singer et al (2006) [22]. These data were used to assign recombination frequencies to windows of 100 Kb.

### Identification of complex insertion events

The expectation was that each FST sequence would contain the T-DNA left border sequence and the adjacent *A. thaliana *genomic sequence. FST sequences that aligned to multiple, non-repetitive genomic regions and/or additional regions of the pSKI015 plasmid indicated multiple, imprecise or complex T-DNA integrations (Figure 3). For each FST the number and orientation of additional plasmid sequence was extracted from the alignment data and used to infer the arrangement of each T-DNA integration event. An example of the sequence alignment data for each different integration event identified is shown in Additional file 3.

### Analysis of genes lacking T-DNA integrations

Considering all five FST datasets (SK, SALK, GABI, SAIL, FLAG), a set of 6,004 AGIs were identified as having no T-DNA insertion. For each gene, six basic gene parameters were examined, including transcript length, expression level, proximity to the centromere, the presence of introns, gene copy number and annotation as a pseudogene. The position of each centromere was taken as: 15,088,987 bp on chromosome 1; 3,608,427 bp on chromosome 2; 13,599,567 bp on chromosome 3; 3,456,519 bp on chromosome 4 and 11,742,755 bp on chromosome 5. Affymetrix gene expression data was analysed as described above, although only 1,913 of the genes lacking an insertion were present on the array. The proximity to the centromere was determined by subtracting the position of the 5' most coordinate of each AGI model from the centromeric coordinate. The distribution for each parameter was not normal according to the Jarque-Bera goodness-of-fit test [56] thus data were analysed according to the non-parametric Wilcoxon-Mann-Whitney test [57]. These data were compared to a distribution of the same size generated by random selection from those genes with insertions.

### Seed Coat Colour and Proanthocyanidin Screens

Variability in seed colour density, colour hue, and proanthocyanidin distribution patterns was observed under a stereo-compound microscope. Particular attention was given to recovering subtle changes in seed colour patterns. Seed colour variants were analyzed for variability in proanthocyanidins and flavan-3-ols using the histochemical stain dimethylaminocinnamylaldehyde (Sigma, Oakville, Canada) (2% DMACA in 3 N HCl 50% [w/v] methanol) [58]. Seed colour variants were grown for three additional generations to confirm trait stability.

### Screening for variation in trichome morphology

Seeds from each line were surface sterilized with chlorine gas and plated onto 1/2 MS media, stratified at 4°C for 3 days in the dark, and then incubated in an Enconair AC-60 at 22°C, under long-day conditions. Trichome morphology was viewed with a Zeiss Stemi 2000-C dissection microscope. Leaves were frozen in liquid nitrogen and micrographs were taken on a JOEL 6400 cry-scanning electron microscope. The average number of branches for trichomes of wild type *A. thaliana *was calculated from 798 plants.

### Additional datasets used

The genome coordinate position data for the FSTs represented in the SALK, FLAGdb, SAIL and GABI-Kat collections were obtained from http://natural.salk.edu/database/transcriptome/T-DNA.SALK, http://natural.salk.edu/database/transcriptome/T-DNA.FLAG, http://natural.salk.edu/database/transcriptome/T-DNA.SAIL and http://natural.salk.edu/database/transcriptome/T-DNA.GABI respectively.

The microarray data used in this study was obtained from slides ATGE_37_A, ATGE_37_B and ATGE_37_C available at http://affymetrix.arabidopsis.info/narrays/experimentpage.pl?experimentid=152.

## Authors' contributions

SJR developed targeted Perl scripts, analysed the FST data, and wrote the manuscript. LHT, BAGM and SJM conducted the plant transformations, selection of transgenic plants, developed the archived T_3 _population by single seed descent and carried out molecular analyses. WEC assisted in the development of targeted Perl scripts, MGL developed software and managed warehousing of the sequence data and SK implemented GBrowse to visualise the FST sites. SR, YYW, MYG and MY utilised the population to identify individuals with variant trichome development. MYG, DC and MY screened the population to identify lines varying in seed coat colour and proanthocyanidin deposition. IAPP designed and coordinated the development of the population, directed and participated in the analysis of results and helped to write the manuscript. Each of the authors have read and approved the final manuscript.

## Supplementary Material

Additional file 1 **The frequency of T-DNA integration is correlated with carpel tissue gene expression level but not recombination rate along the five *A. thaliana *chromosomes**. These graphs demonstrate the relationship among the observed frequency of gene expression, T-DNA integration and genetic recombination observed along each of the five pseudochromosome molecules. Click here for file

Additional file 2 **Statistical analyses studying the impact of gene characteristics on T-DNA integration**. Statistical analyses of the parameters gene expression, gene length, and proximity to the centromere for the two gene classes, those with and without an integration event. Additionally, statistical output are presented that demonstrates the impact of the presence of intron sequence, pseudogene classification and gene redundancy on T-DNA integration. Click here for file

Additional file 3 **Alignment of selected FST sequences to the *A. thaliana *genome**. Blast output showing examples of: A) a simple T-DNA integration event into the *A. thaliana *genome; B) complex integration events described and numbered according to Figure 3. Click here for file
