# Supplementary figures and images for: An archived activation tagged population of Arabidopsis thaliana to facilitate forward genetics approaches
Source: BMC Plant Biol. 2009 Jul 31;9:101. doi: 10.1186/1471-2229-9-101 (PMC3091532; doi:10.1186/1471-2229-9-101)

**Additional file 1.**

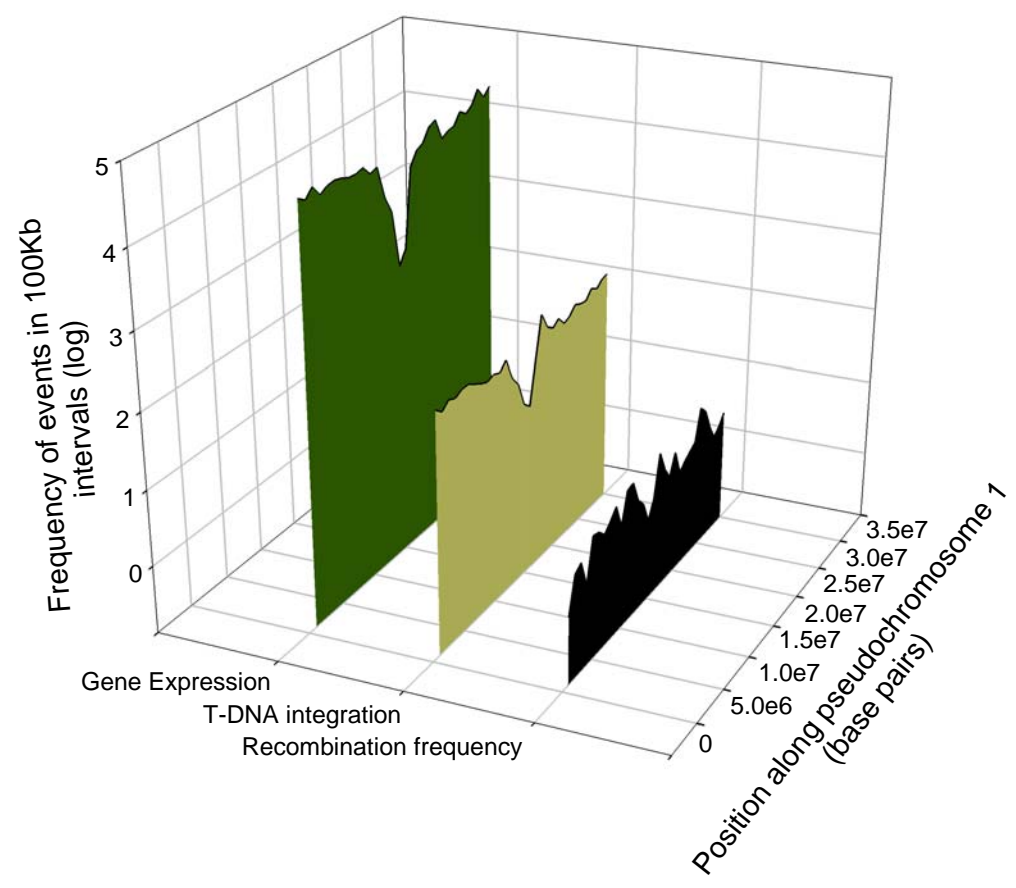

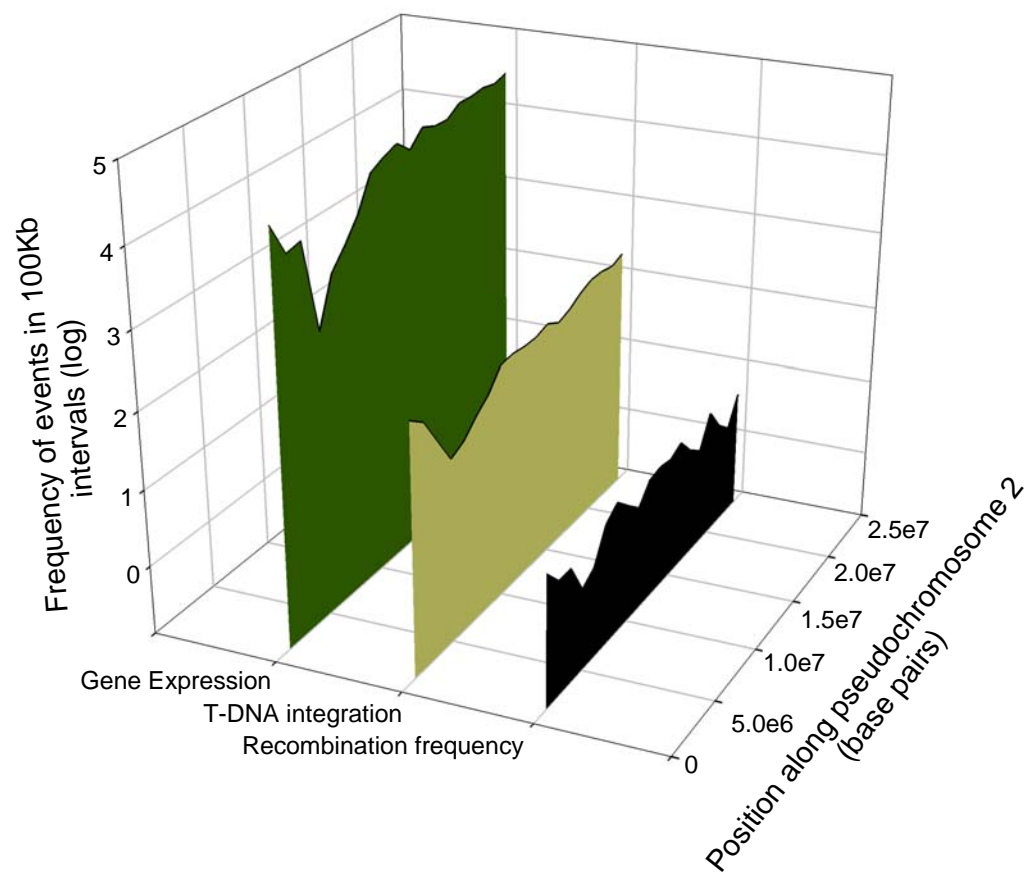

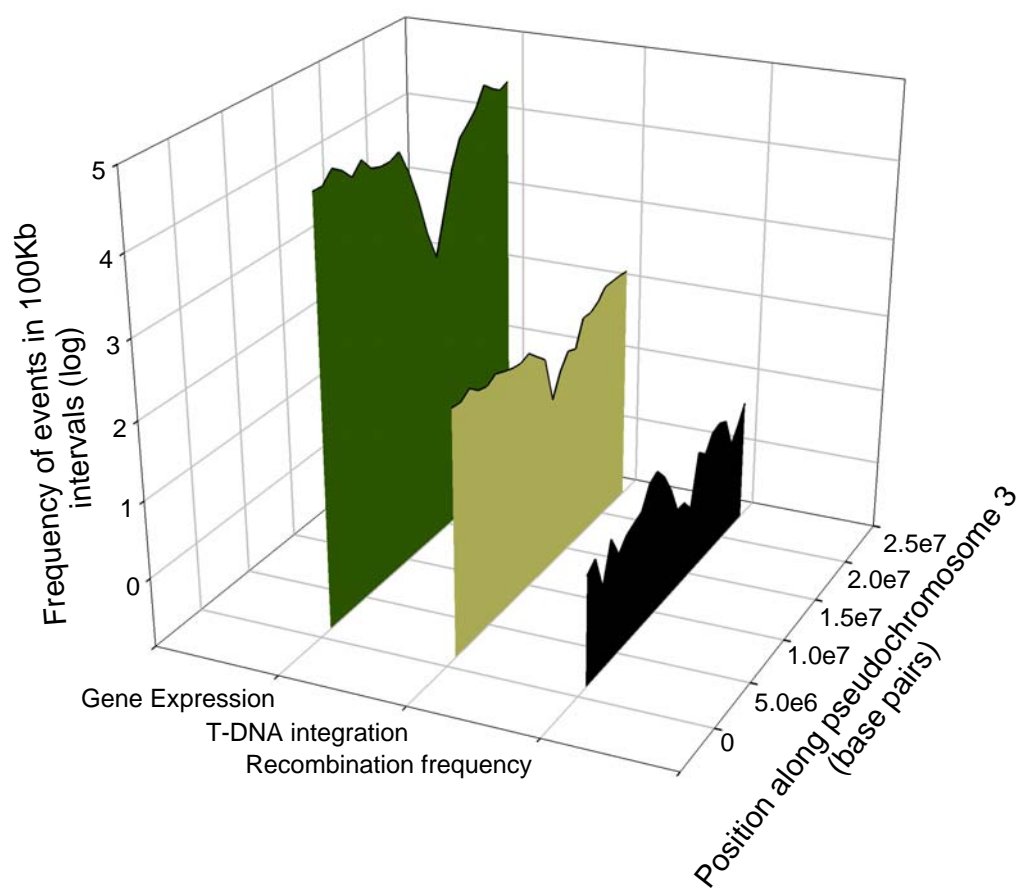

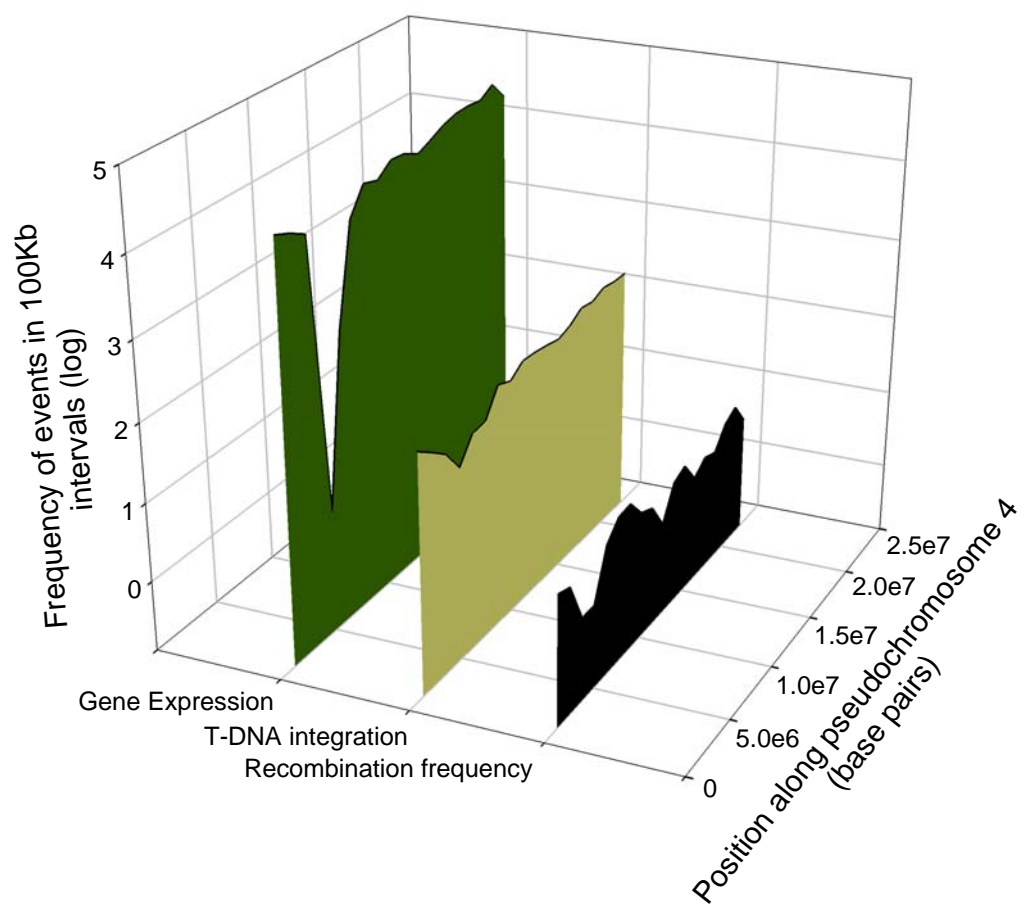

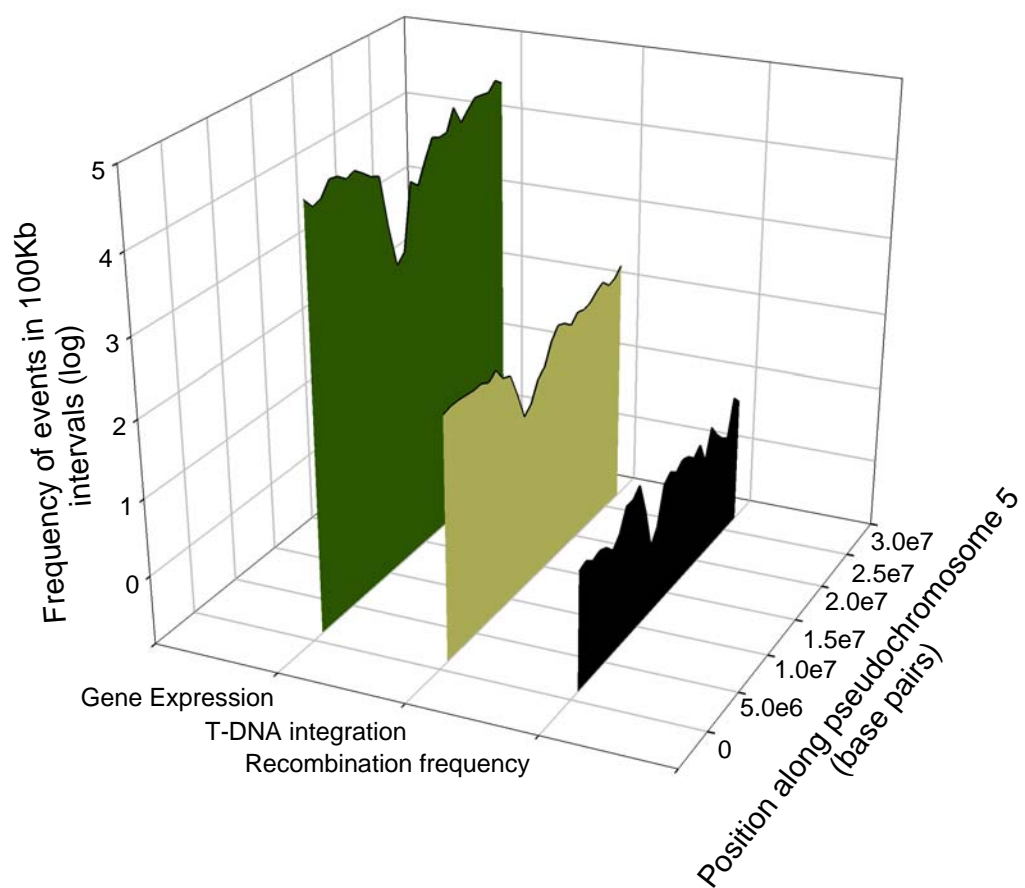

Supplement: Additional file 1 — The frequency of T-DNA integration is correlated with carpel tissue gene expression level but not recombination rate along the five A. thaliana chromosomes. These graphs demonstrate the relationship among the observed frequency of gene expression, T-DNA integration and genetic recombination observed along each of the five pseudochromosome molecules. [file 1471-2229-9-101-S1.pdf]
