# Supplementary material for: An archived activation tagged population of Arabidopsis thaliana to facilitate forward genetics approaches
Source: BMC Plant Biol. 2009 Jul 31;9:101. doi: 10.1186/1471-2229-9-101 (PMC3091532; doi:10.1186/1471-2229-9-101)
Supplement: Additional file 2 — Statistical analyses studying the impact of gene characteristics on T-DNA integration. Statistical analyses of the parameters gene expression, gene length, and proximity to the centromere for the two gene classes, those with and without an integration event. Additionally, statistical output are presented that demonstrates the impact of the presence of intron sequence, pseudogene classification and gene redundancy on T-DNA integration. [file 1471-2229-9-101-S2.pdf]

Additional file 2.

Gene expression level

Genes with no insertion events - None
Genes with insertion events - Present

Data	Mean	Standard Deviation	Median	Upper Quartile	Lower Quartile	n
None	68.175	233.987	7.720	28.653	2.580	1913
Present	125.032	243.122	48.887	131.983	10.350	1913

Jarque-Bera Test:

	JB	Skewness	Kurtosis
None	459095.188	8.393	99.133
Present	119579.022	5.299	40.254

Probability: < 0.0001

Mann-Whitney-Wilcoxon Test:

Dataset	n	Rank sum: observed / expected
1	1913	2909214 /3659569
2	1913	4411836 /3659569

N (size of both datasets): 3826
z: -21.992058
Probability: <0.000001
Ranks of genes with no insertions are lower than expected

Plot of the distributions:

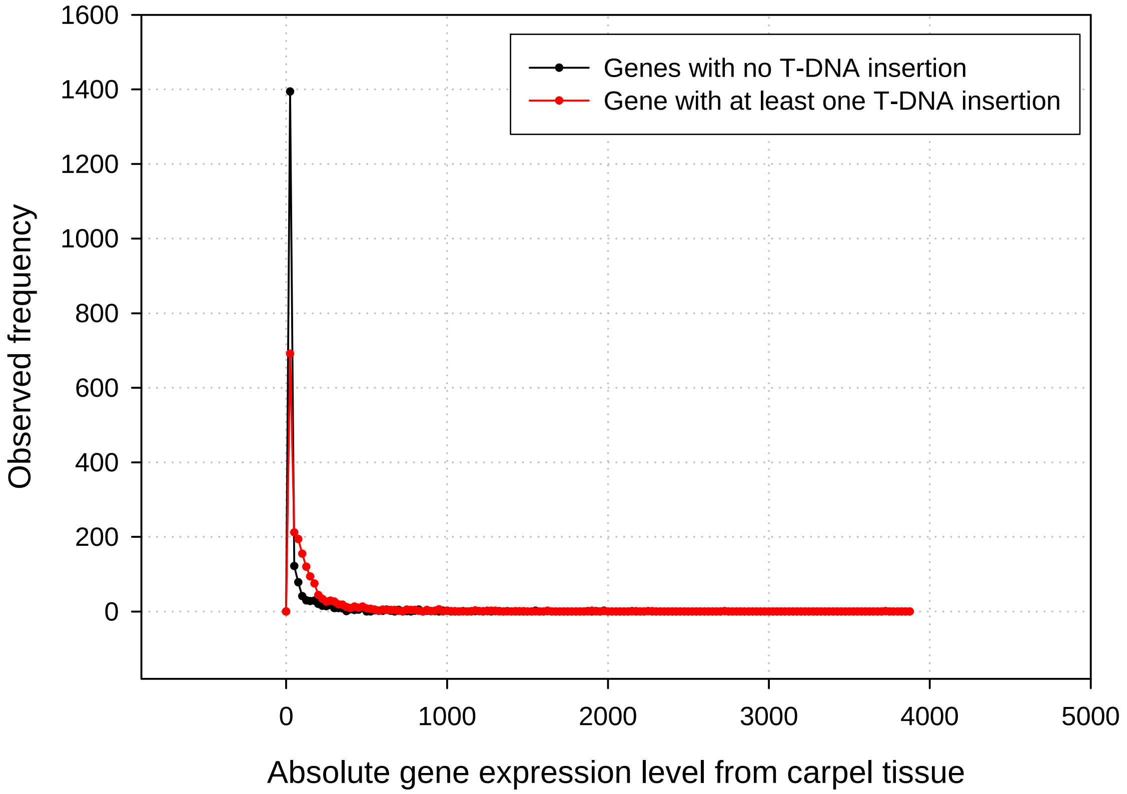

Genes with no T-DNA insertion
Gene with at least one T-DNA insertion
Observed frequency
1600
1400
1200
1000
800
600
400
200
0
0
1000
2000
3000
4000
5000
Absolute gene expression level from carpel tissue

Gene length – transcript (bp)

Genes with no insertion events - None
Genes with insertion events - Present

Data	Mean	Standard Deviation	Median	Upper Quartile	Lower Quartile	n
None	1131.756	911.020	841.000	1467.500	505.000	4806
Present	2418.472	1614.947	2081.500	3037.000	1358.000	4806

Jarque-Bera Test:

	JB	Skewness	Kurtosis
None	11244.068	2.438	8.690
Present	248262.798	4.008	37.285

Probability: < 0.0001

Mann-Whitney-Wilcoxon Test:

Dataset	n	rank sum: observed / expected
1	4806	16145421 /23097636
2	4806	30054657 /23097636

N (size of both datasets): 9612
z: -1048041.30282
Probability: <0.000001
Ranks of genes with no insertions are lower than expected

Plot of the distributions:

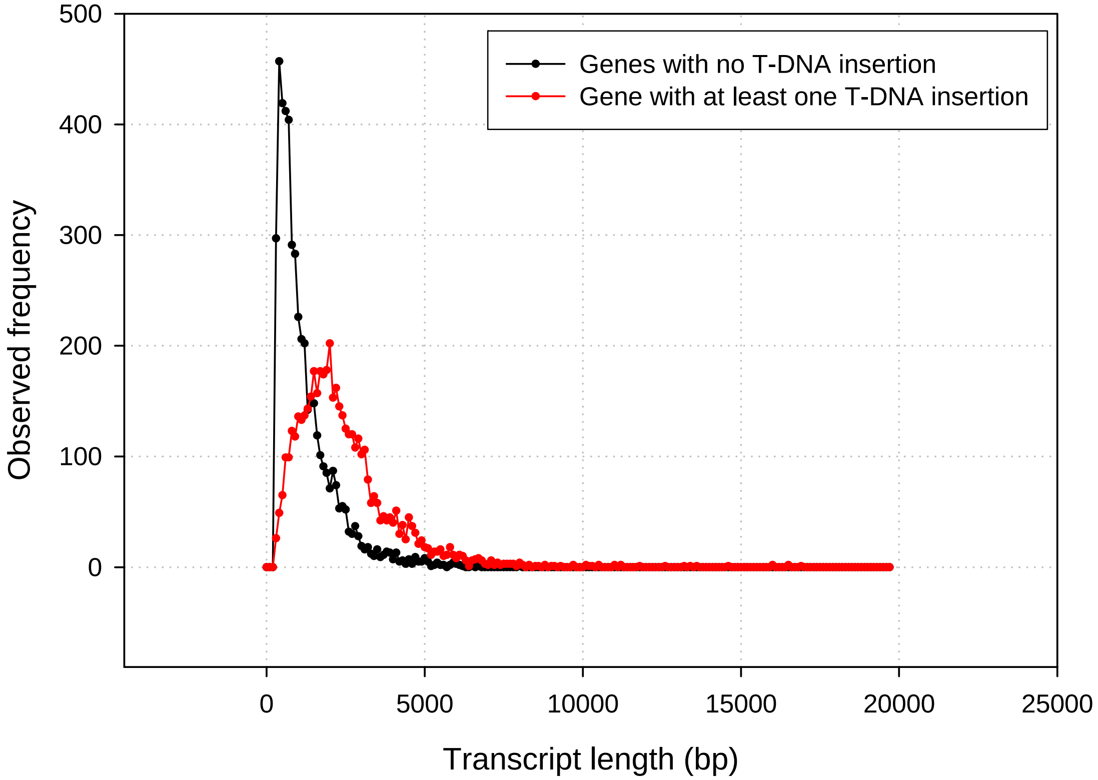

Genes with no T-DNA insertion
Gene with at least one T-DNA insertion
Observed frequency
500
400
300
200
100
0
0
5000
10000
15000
20000
25000
Transcript length (bp)

Position relative to the centromere (Kbp)

Genes with no insertion events - None
Genes with insertion events - Present

Data	Mean	Standard Deviation	Median	Upper Quartile	Lower Quartile	n
None	5069.832	4279.960	3631.0	8115.5	1406127.5	4806
Present	7714.580	4173.393	7803.0	11109.0	4142395.0	4806

Jarque-Bera Test:

	JB	Skewness	Kurtosis
None	1723.144	1.434	2.380
Present	1657.428	1.331	1.910

Probability: < 0.0001

Mann-Whitney-Wilcoxon Test:

Dataset	n	Rank sum: observed / expected
1	4806	18915972 /23097636
2	4806	27284105 /23097636

N (size of both datasets): 9612
z: -30.759262
Probability: <0.000001
Ranks of genes with no insertion are lower than expected

Plot of the distributions:

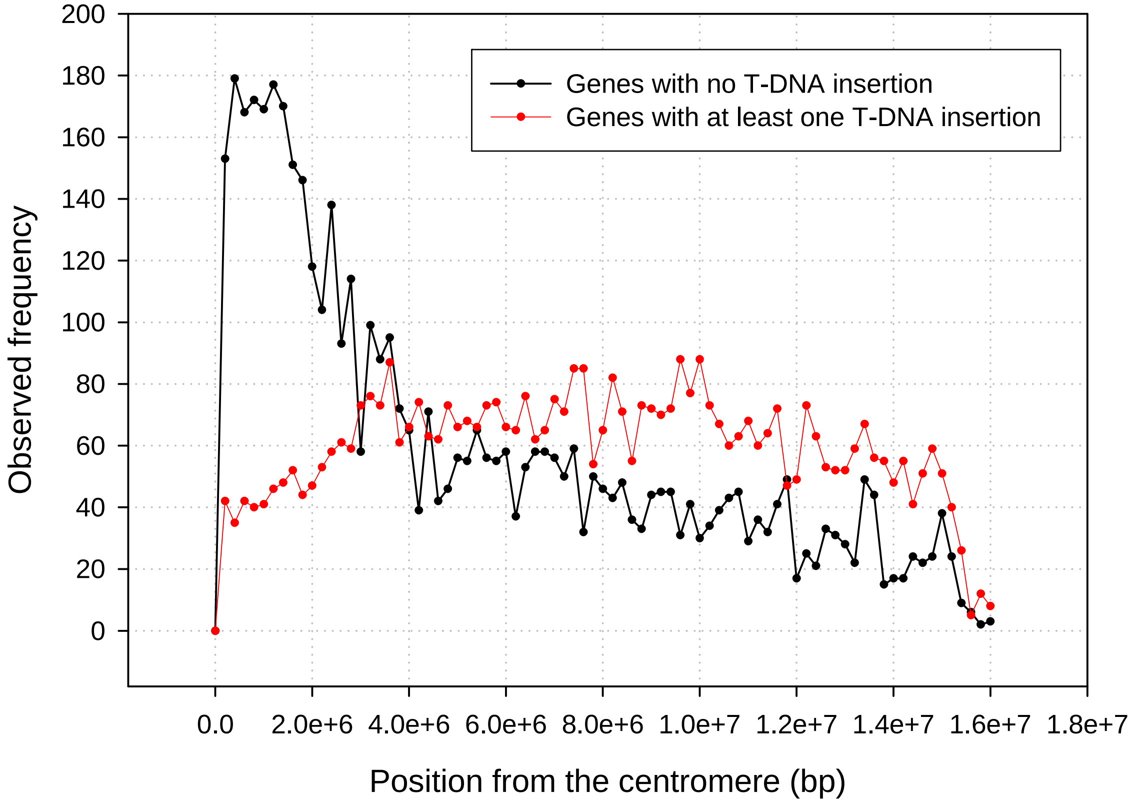

Genes with no T-DNA insertion
Genes with at least one T-DNA insertion
Observed frequency
Position from the centromere (bp)
200
180
160
140
120
100
80
60
40
20
0
0.0
2.0e+6
4.0e+6
6.0e+6
8.0e+6
1.0e+7
1.2e+7
1.4e+7
1.6e+7
1.8e+7

A) χ^2 contingency tables for basic gene characteristics:

i) Intron sequences:

	Genes with T-DNA hit	Genes with no T-DNA hit
Genes with intron	22,940	2,496
Gene without intron	4,338	3,508
$X^2_{\text{1d.f.}}$	4,941	$P < 0.0001$

ii) Annotated pseudogenes:

	Genes with T-DNA hit	Genes with no T-DNA hit
Genes	26,293	5,250
Pseudogenes	985	754
$X^2_{\text{1d.f.}}$	794	$P < 0.0001$

ii) Annotated single copy genes:

	Genes with T-DNA hit	Genes with no T-DNA hit
Gene family	24,140	5,509
Single copy genes	3,138	495
$X^2_{\text{1d.f.}}$	53.8	$P < 0.0001$

B) Proportional analysis of the 6,004 genes without a T-DNA integration

i) Genes lacking intron sequences (23.5% of the total):

	Genes with no introns	Genes with introns
Observed:	2,919	3,085
Expected:	4,593	1,411
$X^2_{\text{1d.f.}}$	2,596.1	$P < 0.0001$

ii) Genes annotated as pseudogenes (5.2% of the total):

	Annotated Genes	Annotated pseudogenes
Observed:	5,250	754
Expected:	5,692	312
$X^2_{\text{1d.f.}}$	660.5	$P < 0.0001$

iii) Genes present as a single copy (11.2% of the total):

	Duplicated Genes	Single Copy Genes
Observed:	5,508	496
Expected:	5,344	660
$X^2_{\text{1d.f.}}$	45.8	$P < 0.0001$
