# Supplementary material for: An archived activation tagged population of Arabidopsis thaliana to facilitate forward genetics approaches
Source: BMC Plant Biol. 2009 Jul 31;9:101. doi: 10.1186/1471-2229-9-101 (PMC3091532; doi:10.1186/1471-2229-9-101)
Supplement: Additional file 3 — Alignment of selected FST sequences to the A. thaliana genome. Blast output showing examples of: A) a simple T-DNA integration event into the A. thaliana genome; B) complex integration events described and numbered according to Figure 3. [file 1471-2229-9-101-S3.pdf]

Additional file 3.

A). FST sequence adjacent to a T-DNA LB

```
BLASTN 2.2.16 [Mar-25-2007]


Reference: Altschul, Stephen F., Thomas L. Madden, Alejandro A. Schaffer,
Jinghui Zhang, Zheng Zhang, Webb Miller, and David J. Lipman (1997),
"Gapped BLAST and PSI-BLAST: a new generation of protein database search
programs",  Nucleic Acids Res. 25:3389-3402.

Query= Skbox-reseq1-1_A15_sk3257_033.ab1 CHROMAT_FILE:
Skbox-reseq1-1_A15_sk3257_033.ab1 PHD_FILE:
Skbox-reseq1-1_A15_sk3257_033.ab1.phd.1 CHEM: term DYE: big TIME: Fri
Mar  2 17:06:28 2007
         (733 letters)

Database: TAIR 8 pseudochromosome database created by Steve on July 15
2008
           7 sequences; 119,707,899 total letters

Searching..................................................done



                                                                 Score    E
Sequences producing significant alignments:                      (bits) Value

Chr1 dumped from ADB: Mar/14/08 12:28; last updated: 2007-12-20      1049   0.0

>Chr1 dumped from ADB: Mar/14/08 12:28; last updated: 2007-12-20
          Length = 30432563

 Score = 1049 bits (529), Expect = 0.0
 Identities = 545/549 (99%), Gaps = 1/549 (0%)
 Strand = Plus / Minus


Query: 87       tgatggtttttttttacgtgagggtgaggcgtgccataaatttagggtcgctttctttaa 146
                ||||||||||||||||||||||||||||||||||||||||||||||||||||||||||||
Sbjct: 27667680 tgatggtttttttttacgtgagggtgaggcgtgccataaatttagggtcgctttctttaa 27667621


Query: 147      ctattttcctcattctaattattacgccttcatatatttattactatcaattttattttt 206
                ||||||||||||||||||||||||||||||||||||||||||||||||||||||||||||
Sbjct: 27667620 ctattttcctcattctaattattacgccttcatatatttattactatcaattttattttt 27667561


Query: 207      atttttgttttgaatacagtcatttcactttctaataaattcatttggtaactaaaataa 266
                |||||||||||| |||||||||||||||||||||||||||||||||||||||||||||||
Sbjct: 27667560 atttttgttttgtatacagtcatttcactttctaataaattcatttggtaactaaaataa 27667501


Query: 267      ataaaaattcatcattccacatatttaaatcaagccaatatttttagcaattaaatgatt 326
                ||||||||||||||||||||||||||||||||||||||||||||||||||||||||||||
Sbjct: 27667500 ataaaaattcatcattccacatatttaaatcaagccaatatttttagcaattaaatgatt 27667441


Query: 327      ttcatgtcgagagtacaattcaaaccaagttaagtattatacatttgatttatcattata 386
                ||||||||||||||||||||||||||||||||||||||||||||||||||||||||||||
Sbjct: 27667440 ttcatgtcgagagtacaattcaaaccaagttaagtattatacatttgatttatcattata 27667381

Query: 387      taagatcgtcgtataaatatgaaggtttgtttttattcataaaaagcatcagcatctaag 446
                ||||||||||||||||||||||||||||||||||||||||||||||||||||||||||||
Sbjct: 27667380 taagatcgtcgtataaatatgaaggtttgtttttattcataaaaagcatcagcatctaag 27667321


Query: 447      gggtatgtaatgattctgagaagaatcggtaaaaggatacacaattgacaacactaaacg 506
                 |||||||||||||||||||||||||||||||||||||||||||||||||||||||||||
Sbjct: 27667320 tggtatgtaatgattctgagaagaatcggtaaaaggatacacaattgacaacactaaacg 27667261


Query: 507      aatctttcaagattccacaaacgcagtgaagaggaatcttgaccaagtcaaataacgtaa 566
                ||||||||||||||||||||||||||||||||||||||||||||||||| ||||||||||
Sbjct: 27667260 aatctttcaagattccacaaacgcagtgaagaggaatcttgaccaagtcgaataacgtaa 27667201
```

```
Query: 567      cgcatgcaccaagtcccctatcttctacatatttaatcgagtgttctgttg-atccaata 625
                ||||||||||||||||||||||||||||||||||||||||||||||||||| ||||||||
Sbjct: 27667200 cgcatgcaccaagtcccctatcttctacatatttaatcgagtgttctgttgaatccaata 27667141


Query: 626      ctcgtgtcg 634
                |||||||||
Sbjct: 27667140 ctcgtgtcg 27667132
```

B). FST sequences indicating complex T-DNA integration

No. 1). Two adjacent insertions LB::RB

```
BLASTN 2.2.16 [Mar-25-2007]


Reference: Altschul, Stephen F., Thomas L. Madden, Alejandro A. Schaffer,
Jinghui Zhang, Zheng Zhang, Webb Miller, and David J. Lipman (1997),
"Gapped BLAST and PSI-BLAST: a new generation of protein database search
programs",  Nucleic Acids Res. 25:3389-3402.

Query= SKboxes93-96-2_F21_sk18105_036.ab1
         (754 letters)

Database: pski015 blast database created by Steve 210708
           1 sequences; 10,138 total letters

Searching..................................................done



                                                                 Score    E
Sequences producing significant alignments:                      (bits) Value

|pski015| sequence of T-DNA used in generation of mutated popul...  1158   0.0

>|pski015| sequence of T-DNA used in generation of mutated population
          Length = 10138

 Score = 1158 bits (584), Expect = 0.0
 Identities = 584/584 (100%)
 Strand = Plus / Minus


Query: 171  tcaaacactgatagtttcggatctagatatcacatcaatccacttgctttgaagacgtgg 230
            ||||||||||||||||||||||||||||||||||||||||||||||||||||||||||||
Sbjct: 4272 tcaaacactgatagtttcggatctagatatcacatcaatccacttgctttgaagacgtgg 4213


Query: 231  ttggaacgtcttctttttccacgatgttcctcgtgggtgggggtccatctttgggaccac 290
            ||||||||||||||||||||||||||||||||||||||||||||||||||||||||||||
Sbjct: 4212 ttggaacgtcttctttttccacgatgttcctcgtgggtgggggtccatctttgggaccac 4153


Query: 291  tgtcggtagaggcatcttgaacgatagcctttcctttatcgcaatgatggcatttgtaga 350
            ||||||||||||||||||||||||||||||||||||||||||||||||||||||||||||
Sbjct: 4152 tgtcggtagaggcatcttgaacgatagcctttcctttatcgcaatgatggcatttgtaga 4093


Query: 351  agccatcttccttttctactgtcctttcgatgaagtgacagatagctgggcaatggaatc 410
            ||||||||||||||||||||||||||||||||||||||||||||||||||||||||||||
Sbjct: 4092 agccatcttccttttctactgtcctttcgatgaagtgacagatagctgggcaatggaatc 4033


Query: 411  cgaggaggtttcccgatattaccctttgttgaaaagtctcaatagccctctggtcttctg 470
            ||||||||||||||||||||||||||||||||||||||||||||||||||||||||||||
Sbjct: 4032 cgaggaggtttcccgatattaccctttgttgaaaagtctcaatagccctctggtcttctg 3973

Query: 471  agactgtatctttgatattcttggagtagacgagagtgtcgtgctccaccatgttgggga 530
            ||||||||||||||||||||||||||||||||||||||||||||||||||||||||||||
Sbjct: 3972 agactgtatctttgatattcttggagtagacgagagtgtcgtgctccaccatgttgggga 3913


Query: 531  tctagatatcacatcaatccacttgctttgaagacgtggttggaacgtcttctttttcca 590
            ||||||||||||||||||||||||||||||||||||||||||||||||||||||||||||
Sbjct: 3912 tctagatatcacatcaatccacttgctttgaagacgtggttggaacgtcttctttttcca 3853
```

```
Query: 591  cgatgttcctcgtgggtgggggtccatctttgggaccactgtcggtagaggcatcttgaa 650
            ||||||||||||||||||||||||||||||||||||||||||||||||||||||||||||
Sbjct: 3852 cgatgttcctcgtgggtgggggtccatctttgggaccactgtcggtagaggcatcttgaa 3793

Query: 651  cgatagcctttcctttatcgcaatgatggcatttgtagaagccatcttccttttctactg 710
            ||||||||||||||||||||||||||||||||||||||||||||||||||||||||||||
Sbjct: 3792 cgatagcctttcctttatcgcaatgatggcatttgtagaagccatcttccttttctactg 3733

Query: 711  tcctttcgatgaagtgacagatagctgggcaatggaatccgagg 754
            ||||||||||||||||||||||||||||||||||||||||||||
Sbjct: 3732 tcctttcgatgaagtgacagatagctgggcaatggaatccgagg 3689
```

Score = 1122 bits (566), Expect = 0.0
Identities = 566/566 (100%)
Strand = Plus / Minus

```
Query: 189  ggatctagatatcacatcaatccacttgctttgaagacgtggttggaacgtcttcttttt 248
            ||||||||||||||||||||||||||||||||||||||||||||||||||||||||||||
Sbjct: 3576 ggatctagatatcacatcaatccacttgctttgaagacgtggttggaacgtcttcttttt 3517

Query: 249  ccacgatgttcctcgtgggtgggggtccatctttgggaccactgtcggtagaggcatctt 308
            ||||||||||||||||||||||||||||||||||||||||||||||||||||||||||||
Sbjct: 3516 ccacgatgttcctcgtgggtgggggtccatctttgggaccactgtcggtagaggcatctt 3457

Query: 309  gaacgatagcctttcctttatcgcaatgatggcatttgtagaagccatcttccttttcta 368
            ||||||||||||||||||||||||||||||||||||||||||||||||||||||||||||
Sbjct: 3456 gaacgatagcctttcctttatcgcaatgatggcatttgtagaagccatcttccttttcta 3397

Query: 369  ctgtcctttcgatgaagtgacagatagctgggcaatggaatccgaggaggtttcccgata 428
            ||||||||||||||||||||||||||||||||||||||||||||||||||||||||||||
Sbjct: 3396 ctgtcctttcgatgaagtgacagatagctgggcaatggaatccgaggaggtttcccgata 3337

Query: 429  ttaccctttgttgaaaagtctcaatagccctctggtcttctgagactgtatctttgatat 488
            ||||||||||||||||||||||||||||||||||||||||||||||||||||||||||||
Sbjct: 3336 ttaccctttgttgaaaagtctcaatagccctctggtcttctgagactgtatctttgatat 3277

Query: 489  tcttggagtagacgagagtgtcgtgctccaccatgttggggatctagatatcacatcaat 548
            ||||||||||||||||||||||||||||||||||||||||||||||||||||||||||||
Sbjct: 3276 tcttggagtagacgagagtgtcgtgctccaccatgttggggatctagatatcacatcaat 3217

Query: 549  ccacttgctttgaagacgtggttggaacgtcttcttttttccacgatgttcctcgtgggtg 608
            ||||||||||||||||||||||||||||||||||||||||||||||||||||||||||||
Sbjct: 3216 ccacttgctttgaagacgtggttggaacgtcttcttttttccacgatgttcctcgtgggtg 3157

Query: 609  ggggtccatctttgggaccactgtcggtagaggcatcttgaacgatagcctttcctttat 668
            ||||||||||||||||||||||||||||||||||||||||||||||||||||||||||||
Sbjct: 3156 ggggtccatctttgggaccactgtcggtagaggcatcttgaacgatagcctttcctttat 3097

Query: 669  cgcaatgatggcatttgtagaagccatcttccttttctactgtcctttcgatgaagtgac 728
            ||||||||||||||||||||||||||||||||||||||||||||||||||||||||||||
Sbjct: 3096 cgcaatgatggcatttgtagaagccatcttccttttctactgtcctttcgatgaagtgac 3037

Query: 729  agatagctgggcaatggaatccgagg 754
            ||||||||||||||||||||||||||
Sbjct: 3036 agatagctgggcaatggaatccgagg 3011
```

Score = 1122 bits (566), Expect = 0.0
Identities = 566/566 (100%)
Strand = Plus / Minus

```
Query: 189  ggatctagatatcacatcaatccacttgctttgaagacgtggttggaacgtcttcttttt 248
            ||||||||||||||||||||||||||||||||||||||||||||||||||||||||||||
Sbjct: 3915 ggatctagatatcacatcaatccacttgctttgaagacgtggttggaacgtcttcttttt 3856
```

```
Query: 249  ccacgatgttcctcgtgggtgggggtccatctttgggaccactgtcggtagaggcatctt 308
            ||||||||||||||||||||||||||||||||||||||||||||||||||||||||||||
Sbjct: 3855 ccacgatgttcctcgtgggtgggggtccatctttgggaccactgtcggtagaggcatctt 3796


Query: 309  gaacgatagcctttcctttatcgcaatgatggcatttgtagaagccatcttccttttcta 368
            ||||||||||||||||||||||||||||||||||||||||||||||||||||||||||||
Sbjct: 3795 gaacgatagcctttcctttatcgcaatgatggcatttgtagaagccatcttccttttcta 3736


Query: 369  ctgtcctttcgatgaagtgacagatagctgggcaatggaatccgaggaggtttcccgata 428
            ||||||||||||||||||||||||||||||||||||||||||||||||||||||||||||
Sbjct: 3735 ctgtcctttcgatgaagtgacagatagctgggcaatggaatccgaggaggtttcccgata 3676


Query: 429  ttaccctttgttgaaaagtctcaatagccctctggtcttctgagactgtatctttgatat 488
            ||||||||||||||||||||||||||||||||||||||||||||||||||||||||||||
Sbjct: 3675 ttaccctttgttgaaaagtctcaatagccctctggtcttctgagactgtatctttgatat 3616


Query: 489  tcttggagtagacgagagtgtcgtgctccaccatgttggggatctagatatcacatcaat 548
            ||||||||||||||||||||||||||||||||||||||||||||||||||||||||||||
Sbjct: 3615 tcttggagtagacgagagtgtcgtgctccaccatgttggggatctagatatcacatcaat 3556


Query: 549  ccacttgctttgaagacgtggttggaacgtcttctttttccacgatgttcctcgtgggtg 608
            ||||||||||||||||||||||||||||||||||||||||||||||||||||||||||||
Sbjct: 3555 ccacttgctttgaagacgtggttggaacgtcttctttttccacgatgttcctcgtgggtg 3496


Query: 609  ggggtccatctttgggaccactgtcggtagaggcatcttgaacgatagcctttcctttat 668
            ||||||||||||||||||||||||||||||||||||||||||||||||||||||||||||
Sbjct: 3495 ggggtccatctttgggaccactgtcggtagaggcatcttgaacgatagcctttcctttat 3436


Query: 669  cgcaatgatggcatttgtagaagccatcttccttttctactgtcctttcgatgaagtgac 728
            ||||||||||||||||||||||||||||||||||||||||||||||||||||||||||||
Sbjct: 3435 cgcaatgatggcatttgtagaagccatcttccttttctactgtcctttcgatgaagtgac 3376


Query: 729  agatagctgggcaatggaatccgagg 754
            ||||||||||||||||||||||||||
Sbjct: 3375 agatagctgggcaatggaatccgagg 3350


 Score =  682 bits (344), Expect = 0.0
 Identities = 344/344 (100%)
 Strand = Plus / Minus


Query: 189  ggatctagatatcacatcaatccacttgctttgaagacgtggttggaacgtcttcttttt 248
            ||||||||||||||||||||||||||||||||||||||||||||||||||||||||||||
Sbjct: 3237 ggatctagatatcacatcaatccacttgctttgaagacgtggttggaacgtcttcttttt 3178


Query: 249  ccacgatgttcctcgtgggtgggggtccatctttgggaccactgtcggtagaggcatctt 308
            ||||||||||||||||||||||||||||||||||||||||||||||||||||||||||||
Sbjct: 3177 ccacgatgttcctcgtgggtgggggtccatctttgggaccactgtcggtagaggcatctt 3118


Query: 309  gaacgatagcctttcctttatcgcaatgatggcatttgtagaagccatcttccttttcta 368
            ||||||||||||||||||||||||||||||||||||||||||||||||||||||||||||
Sbjct: 3117 gaacgatagcctttcctttatcgcaatgatggcatttgtagaagccatcttccttttcta 3058


Query: 369  ctgtcctttcgatgaagtgacagatagctgggcaatggaatccgaggaggtttcccgata 428
            ||||||||||||||||||||||||||||||||||||||||||||||||||||||||||||
Sbjct: 3057 ctgtcctttcgatgaagtgacagatagctgggcaatggaatccgaggaggtttcccgata 2998


Query: 429  ttaccctttgttgaaaagtctcaatagccctctggtcttctgagactgtatctttgatat 488
            ||||||||||||||||||||||||||||||||||||||||||||||||||||||||||||
Sbjct: 2997 ttaccctttgttgaaaagtctcaatagccctctggtcttctgagactgtatctttgatat 2938


Query: 489  tcttggagtagacgagagtgtcgtgctccaccatgttggggatc 532
            ||||||||||||||||||||||||||||||||||||||||||||
Sbjct: 2937 tcttggagtagacgagagtgtcgtgctccaccatgttggggatc 2894
```

```
 Score =  256 bits (129), Expect = 6e-71
 Identities = 141/145 (97%)
 Strand = Plus / Minus


Query: 18   attttataataacgtcgcggacatcgacattttgaattgaaaaaacattggtaattact 77
            ||||||||||||||  ||||||||| ||||||||||||||||||| |||||||||||||
Sbjct: 7852 attttataataacgctgcggacatctacattttgaattgaaaaaaaattggtaattact 7793


Query: 78   ctttcttttcctccatattgaccatcatactcattgctgatccatgtagatttcccggac 137
            ||||||||||||||||||||||||||||||||||||||||||||||||||||||||||||
Sbjct: 7792 ctttcttttcctccatattgaccatcatactcattgctgatccatgtagatttcccggac 7733


Query: 138  atgaagccatttacaattgaatata 162
            |||||||||||||||||||||||||
Sbjct: 7732 atgaagccatttacaattgaatata 7708
```

No. 2). Two independent insertions – left border

```
BLASTN 2.2.16 [Mar-25-2007]


Reference: Altschul, Stephen F., Thomas L. Madden, Alejandro A. Schaffer,
Jinghui Zhang, Zheng Zhang, Webb Miller, and David J. Lipman (1997),
"Gapped BLAST and PSI-BLAST: a new generation of protein database search
programs",  Nucleic Acids Res. 25:3389-3402.

Query= SKboxes159-162-1_G13_sk24196_014.ab1 CHROMAT_FILE:
SKboxes159-162-1_G13_sk24196_014.ab1 PHD_FILE:
SKboxes159-162-1_G13_sk24196_014.ab1.phd.1 CHEM: term DYE: big TIME:
Fri Mar  2 16:34:59 2007
         (731 letters)

Database: TAIR 8 pseudochromosome database created by Steve on July 15
2008
           7 sequences; 119,707,899 total letters

Searching..................................................done



                                                                 Score    E
Sequences producing significant alignments:                      (bits) Value

Chr2 CHROMOSOME dumped from ADB: Mar/14/08 12:28; last updated: ...   256   6e-67
Chr5 CHROMOSOME dumped from ADB: Mar/14/08 12:29; last updated: ...   228   1e-58

>Chr2 CHROMOSOME dumped from ADB: Mar/14/08 12:28; last updated: 2007-12-20
          Length = 19705359

 Score =  256 bits (129), Expect = 6e-67
 Identities = 141/145 (97%)
 Strand = Plus / Minus


Query: 176     atcccttgggatgacatgcgttttcggggttttgtttctcgatcattaattagattctag 235
               ||||||||||||||||||||||||||||||||||||||||||||||||||||| ||||||
Sbjct: 7902094 atcccttgggatgacatgcgttttcggggttttgtttctcgatcattaattagtttctag 7902035


Query: 236     aaagagagacgacgtcaagagtcaaaaccaaatgaaaggaggaaccacacgcgcgttgaa 295
               |||||||||||||||||||||||||||||||||||||| ||||||||||| |||||||||
Sbjct: 7902034 aaagagagacgacgtcaagagtcaaaaccaaatgaaagtaggaaccacacacgcgttgaa 7901975


Query: 296     atgttcatagatttcttcacctttg 320
               |||||||||| ||||||||||||||
Sbjct: 7901974 atgttcatagttttcttcacctttg 7901950


>Chr5 CHROMOSOME dumped from ADB: Mar/14/08 12:29; last updated: 2007-12-20
          Length = 26992728
```

```
 Score =  228 bits (115), Expect = 1e-58
 Identities = 156/169 (92%), Gaps = 1/169 (0%)
 Strand = Plus / Minus


Query: 334      gagnaagngaacatcatgaagccattaccataccgaacacgcacatgcatattattattt 393
                ||| ||| ||||||||||||||||||||||||||||||||||||||||||||||||||||
Sbjct: 26368635 gagtaagtgaacatcatgaagccattaccataccgaacacgcacatgcatattattattt 26368576


Query: 394      ataaaattccgctaattgagtttttgtatatacttatttaatgtcgacaagaaaaaaaaa 453
                |||||||| |||||||||||||||||||||||||||||||||||||||||||||||||||
Sbjct: 26368575 ataaaatt-cgctaattgagtttttgtatatacttatttaatgtcgacaagaaaaaaaaa 26368517


Query: 454      aaatccaaggagggtcctcggtgggtaaatcagtcggttttggggaaat 502
                | |||||| ||| || ||  || ||| |||||||| ||||||| |||||
Sbjct: 26368516 atatccaatgagtgttcttcgttggttaatcagtctgttttggtgaaat 26368468
```

No. 3). Two adjacent insertions – RB::RB

```
BLASTN 2.2.16 [Mar-25-2007]


Reference: Altschul, Stephen F., Thomas L. Madden, Alejandro A. Schaffer,
Jinghui Zhang, Zheng Zhang, Webb Miller, and David J. Lipman (1997),
"Gapped BLAST and PSI-BLAST: a new generation of protein database search
programs",  Nucleic Acids Res. 25:3389-3402.

Query= SKbox238-241_O24_sk32837_102.ab1 CHROMAT_FILE:
SKbox238-241_O24_sk32837_102.ab1 PHD_FILE:
SKbox238-241_O24_sk32837_102.ab1.phd.1 CHEM: term DYE: big TIME: Fri
Mar  2 16:15:07 2007
         (756 letters)

Database: TAIR 8 pseudochromosome database created by Steve on July 15
2008
           7 sequences; 119,707,899 total letters

Searching..................................................done



                                                                 Score    E
Sequences producing significant alignments:                      (bits) Value

Chr2 CHROMOSOME dumped from ADB: Mar/14/08 12:28; last updated: ...   297   2e-79

>Chr2 CHROMOSOME dumped from ADB: Mar/14/08 12:28; last updated: 2007-12-20
          Length = 19705359

 Score =  297 bits (150), Expect = 2e-79
 Identities = 208/226 (92%), Gaps = 1/226 (0%)
 Strand = Plus / Plus


Query: 176      gaagcaattcttttcatttcatttatagttggattgtgtttggcgttcaattactttacg 235
                |||||||||| |||| |||||||||| ||||||||||||||||||||||||||| |||||
Sbjct: 10399817 gaagcaattcgtttcgtttcatttattgttggattgtgtttggcgttcaattacgttacg 10399876


Query: 236      gattctatgaatgaagataaaacatttagttacaatgtggtaatacaagcttcacatgac 295
                ||||  |||||||||||||||||||||||||||||||||||||||||||||||||| |||
Sbjct: 10399877 gattggatgaatgaagataaaacatttagttacaatgtggtaatacaagcttcacaggac 10399936


Query: 296      agaacatcacactttcattgtctacttataaatgattgacgtacaataatgtgagtagaa 355
                |||||||||||||||||||||||| | |||||||||||| ||||||||||||||||||||
Sbjct: 10399937 agaacatcacactttcattgtctaatgataaatgattgaggtacaataatgtgagtagaa 10399996


Query: 356      atttttactgtctttgaggccgtgcg-atcaccattgcctctgtct 400
                ||| ||| |||||||||  |  | || ||||||||||||||||||
Sbjct: 10399997 attgttagtgtctttgatccacttcgtatcaccattgcctctgtct 10400042


Score =  105 bits (53), Expect = 1e-21
```

```
 Identities = 86/97 (88%)
 Strand = Plus / Minus


Query: 448      aagaacattaaatctaacattttcttattgtacactaattcactaacaatcagatctgat 507
                |||||||| ||| | ||||||||||||||||||||  | ||||||||||||||||||| |
Sbjct: 10399447 aagaacatgaaagcaaacattttcttattgtacacctagtcactaacaatcagatctgct 10399388


Query: 508      agtatgttcttatttaaaacaacttactactcaactt 544
                ||| ||||||||||| ||||||||||  |||||||||
Sbjct: 10399387 agtttgttcttatttcaaacaacttagcactcaactt 10399351
```

No. 4). Two insertions nested – in left border

```
BLASTN 2.2.16 [Mar-25-2007]


Reference: Altschul, Stephen F., Thomas L. Madden, Alejandro A. Schaffer,
Jinghui Zhang, Zheng Zhang, Webb Miller, and David J. Lipman (1997),
"Gapped BLAST and PSI-BLAST: a new generation of protein database search
programs",  Nucleic Acids Res. 25:3389-3402.

Query= SKbox298_O13_sk39195_015.ab1
         (760 letters)

Database: pski015 blast database created by Steve 210708
           1 sequences; 10,138 total letters

Searching.................................................done



                                                                 Score    E
Sequences producing significant alignments:                      (bits) Value

|pski015| sequence of T-DNA used in generation of mutated popul...   149   9e-39

>|pski015| sequence of T-DNA used in generation of mutated population
          Length = 10138

 Score =  149 bits (75), Expect = 9e-39
 Identities = 84/87 (96%)
 Strand = Plus / Minus


Query: 38   ataataaagctgcggacatctacatttatgaattgaaaaaaaattggtaattactctttc 97
            ||||||| ||||||||||||||||||| ||||||||||||||||||||||||||||||||
Sbjct: 7847 ataataacgctgcggacatctacattttgaattgaaaaaaaattggtaattactctttc 7788


Query: 98   ttttcctccatatagaccatcatactc 124
            ||||||||||||| |||||||||||||
Sbjct: 7787 ttttcctccatattgaccatcatactc 7761



 Score = 99.6 bits (50), Expect = 8e-24
 Identities = 56/58 (96%)
 Strand = Plus / Minus


Query: 140  gaattgaaaaaaaattggtaattactctttcttttccaccatattgaccatcttactc 197
            ||||||||||||||||||||||||||||||||||||| |||||||||||||| |||||
Sbjct: 7818 gaattgaaaaaaaattggtaattactctttcttttcctccatattgaccatcatactc 7761
```

No. 5). Two adjacent insertions – LB::LB

```
BLASTN 2.2.16 [Mar-25-2007]


Reference: Altschul, Stephen F., Thomas L. Madden, Alejandro A. Schaffer,
Jinghui Zhang, Zheng Zhang, Webb Miller, and David J. Lipman (1997),
```

```
"Gapped BLAST and PSI-BLAST: a new generation of protein database search
programs",  Nucleic Acids Res. 25:3389-3402.

Query= SKbox242-245_E14_sk33079_019.ab1
         (768 letters)

Database: pski015 blast database created by Steve 210708
           1 sequences; 10,138 total letters

Searching..................................................done



                                                                 Score    E
Sequences producing significant alignments:                      (bits) Value

|pski015| sequence of T-DNA used in generation of mutated popul...   182   7e-49

>|pski015| sequence of T-DNA used in generation of mutated population
          Length = 10138

 Score =  182 bits (92), Expect = 7e-49
 Identities = 92/92 (100%)
 Strand = Plus / Minus


Query: 70   aaattggtaattactctttcttttcctccatattgaccatcatactcattgctgatccat 129
            ||||||||||||||||||||||||||||||||||||||||||||||||||||||||||||
Sbjct: 7807 aaattggtaattactctttcttttcctccatattgaccatcatactcattgctgatccat 7748


Query: 130  gtagatttcccggacatgaagccatttacaat 161
            ||||||||||||||||||||||||||||||||
Sbjct: 7747 gtagatttcccggacatgaagccatttacaat 7716



 Score =  174 bits (88), Expect = 2e-46
 Identities = 118/128 (92%)
 Strand = Plus / Plus


Query: 180  ggatatattcaattgtaaatggcttcatgtacgggaaatctacatggatcagcaatgagt 239
            |||||||||||||||||||||||||||||| |||||||||||||||||||||||||||||
Sbjct: 7705 ggatatattcaattgtaaatggcttcatgtccgggaaatctacatggatcagcaatgagt 7764


Query: 240  atgatggtcaagatggaggaaaagaaagagttttcccaattttttttatcattcatcaat 299
            ||||||||||| |||||||||||||||||||  || |||||||||||   |||||  |||
Sbjct: 7765 atgatggtcaatatggaggaaaagaaagagtaattaccaattttttttcaattcaaaaat 7824


Query: 300  gtagatgt 307
            ||||||||
Sbjct: 7825 gtagatgt 7832
```

No. 6). Three insertions – two nested in a third

```
BLASTN 2.2.16 [Mar-25-2007]


Reference: Altschul, Stephen F., Thomas L. Madden, Alejandro A. Schaffer,
Jinghui Zhang, Zheng Zhang, Webb Miller, and David J. Lipman (1997),
"Gapped BLAST and PSI-BLAST: a new generation of protein database search
programs",  Nucleic Acids Res. 25:3389-3402.

Query= SKboxes250-253_I10_sk33850_053.ab1
         (746 letters)

Database: pski015 blast database created by Steve 210708
           1 sequences; 10,138 total letters

Searching..................................................done



                                                                 Score    E
Sequences producing significant alignments:                      (bits) Value
```

```
|pski015| sequence of T-DNA used in generation of mutated popul...   206   5e-56

>|pski015| sequence of T-DNA used in generation of mutated population
          Length = 10138

 Score =  206 bits (104), Expect = 5e-56
 Identities = 107/108 (99%)
 Strand = Plus / Minus


Query: 159  aaacactgatagtttcggatctagatatcacatcaatccacttgctttgaagacgtggtt 218
            ||||||||||||||||||||||||||||||||||||||||||||||||||||||||||||
Sbjct: 4270 aaacactgatagtttcggatctagatatcacatcaatccacttgctttgaagacgtggtt 4211


Query: 219  ggaacgtcttctttttccacgatgttcctcgtgggtgggggttcatct 266
            |||||||||||||||||||||||||||||||||||||||||| |||||
Sbjct: 4210 ggaacgtcttctttttccacgatgttcctcgtgggtgggggtccatct 4163



 Score =  174 bits (88), Expect = 2e-46
 Identities = 91/92 (98%)
 Strand = Plus / Minus


Query: 175  ggatctagatatcacatcaatccacttgctttgaagacgtggttggaacgtcttcttttt 234
            ||||||||||||||||||||||||||||||||||||||||||||||||||||||||||||
Sbjct: 3237 ggatctagatatcacatcaatccacttgctttgaagacgtggttggaacgtcttcttttt 3178


Query: 235  ccacgatgttcctcgtgggtgggggttcatct 266
            |||||||||||||||||||||||||| |||||
Sbjct: 3177 ccacgatgttcctcgtgggtgggggtccatct 3146



 Score =  174 bits (88), Expect = 2e-46
 Identities = 91/92 (98%)
Strand = Plus / Minus


Query: 175  ggatctagatatcacatcaatccacttgctttgaagacgtggttggaacgtcttcttttt 234
            ||||||||||||||||||||||||||||||||||||||||||||||||||||||||||||
Sbjct: 3576 ggatctagatatcacatcaatccacttgctttgaagacgtggttggaacgtcttcttttt 3517


Query: 235  ccacgatgttcctcgtgggtgggggttcatct 266
            |||||||||||||||||||||||||| |||||
Sbjct: 3516 ccacgatgttcctcgtgggtgggggtccatct 3485



 Score =  174 bits (88), Expect = 2e-46
 Identities = 91/92 (98%)
 Strand = Plus / Minus


Query: 175  ggatctagatatcacatcaatccacttgctttgaagacgtggttggaacgtcttcttttt 234
            ||||||||||||||||||||||||||||||||||||||||||||||||||||||||||||
Sbjct: 3915 ggatctagatatcacatcaatccacttgctttgaagacgtggttggaacgtcttcttttt 3856


Query: 235  ccacgatgttcctcgtgggtgggggttcatct 266
            |||||||||||||||||||||||||| |||||
Sbjct: 3855 ccacgatgttcctcgtgggtgggggtccatct 3824



 Score = 93.7 bits (47), Expect = 5e-22
 Identities = 84/95 (88%), Gaps = 1/95 (1%)
 Strand = Plus / Minus


Query: 55   cattttgaattgaatcaaa-ttggtagttactctttcttttcctccatatagaccatca 113
            |||||||||||||||  ||| |||||| ||||||||||||||||||||||| ||||||||
Sbjct: 7825 cattttgaattgaaaaaaaaattggtaattactctttcttttcctccatattgaccatca 7766


Query: 114  tactctctgctcatccacaaagatttcccggacat 148
```

```
            |||||  |||| |||||   |||||||||||||||
Sbjct: 7765 tactcattgctgatccatgtagatttcccggacat 7731



 Score = 67.9 bits (34), Expect = 3e-14
 Identities = 40/42 (95%)
 Strand = Plus / Minus


Query: 412  gtaatttgttgttatatcaaaatgtactttcattttataata 453
            ||||||||| ||| ||||||||||||||||||||||||||||
Sbjct: 7883 gtaatttgtcgttttatcaaaatgtactttcattttataata 7842


Score = 52.0 bits (26), Expect = 2e-09
 Identities = 53/62 (85%)
 Strand = Plus / Minus


Query: 293  acttacattttataatgacgatgcggactttttattttggaattgaaaaaaaatgggta 352
            |||| |||||||||||| ||| ||||||| | |  ||||| |||||||||||||| ||||
Sbjct: 7858 actttcattttataataacgctgcggacatctacattttgaattgaaaaaaaattggta 7799


Query: 353  at 354
            ||
Sbjct: 7798 at 7797
```

No. 7). Two insertions with replicated LB sequence

```
BLASTN 2.2.16 [Mar-25-2007]


Reference: Altschul, Stephen F., Thomas L. Madden, Alejandro A. Schaffer,
Jinghui Zhang, Zheng Zhang, Webb Miller, and David J. Lipman (1997),
"Gapped BLAST and PSI-BLAST: a new generation of protein database search
programs",  Nucleic Acids Res. 25:3389-3402.

Query= SKbox315-318_C06_sk40665_089.ab1
         (662 letters)

Database: pski015 blast database created by Steve 210708
           1 sequences; 10,138 total letters

Searching..................................................done



                                                                 Score    E
Sequences producing significant alignments:                      (bits) Value

|pski015| sequence of T-DNA used in generation of mutated popul...   303   2e-85

>|pski015| sequence of T-DNA used in generation of mutated population
          Length = 10138

 Score =  303 bits (153), Expect = 2e-85
 Identities = 216/236 (91%), Gaps = 1/236 (0%)
 Strand = Plus / Minus


Query: 193  aataaagatttccgaattagaataatttgtttatgtgctttcgcctatccatacgacgga 252
            |||||||||||||||||||||||||||||||||| |||||||||||||  ||||||||||
Sbjct: 7944 aataaagatttccgaattagaataatttgtttat-tgctttcgcctataaatacgacgga 7886


Query: 253  tcgtaatttgtcgttttgtcaaaatgaactttcattttataataacgctgcggacgtcta 312
            ||||||||||||||||| |||||||| |||||||||||||||||||||||||||| ||||
Sbjct: 7885 tcgtaatttgtcgttttatcaaaatgtactttcattttataataacgctgcggacatcta 7826


Query: 313  cattttgaattgaaaaagaattgggaattgcgctttcttttcctccgtgttgaccatca 372
            |||||||||||||||||| |||||| |||| | |||||||||||||| | ||||||||||
Sbjct: 7825 cattttgaattgaaaaaaaattggtaattactctttcttttcctccatattgaccatca 7766


Query: 373  tactcgttgctcatgcgtggagatttcccggacatgaagccctttaccnttgaata 428
            ||||| ||||| || | || |||||||||||||||||||| |||||  |||||||
```

```
Sbjct: 7765 tactcattgctgatccatgtagatttcccggacatgaagccatttacaattgaata 7710


 Score =  153 bits (77), Expect = 5e-40
 Identities = 84/85 (98%), Gaps = 1/85 (1%)
 Strand = Plus / Minus


Query: 11   ctctttcttttcctc-atattgaccatcatactcattgctgatccatgtagatttcccgg 69
            ||||||||||||||| ||||||||||||||||||||||||||||||||||||||||||||
Sbjct: 7794 ctctttcttttcctccatattgaccatcatactcattgctgatccatgtagatttcccgg 7735


Query: 70   acatgaagccatttacaattgaata 94
            |||||||||||||||||||||||||
Sbjct: 7734 acatgaagccatttacaattgaata 7710


 Score =  151 bits (76), Expect = 2e-39
 Identities = 76/76 (100%)
 Strand = Plus / Minus


Query: 586  aaataaagatttccgaattagaataatttgtttattgctttcgcctataaatacgacgga 645
            ||||||||||||||||||||||||||||||||||||||||||||||||||||||||||||
Sbjct: 7945 aaataaagatttccgaattagaataatttgtttattgctttcgcctataaatacgacgga 7886


Query: 646  tcgtaatttgtcgttt 661
            ||||||||||||||||
Sbjct: 7885 tcgtaatttgtcgttt 7870
```

No. 8). Two insertions nested in Ti backbone

```
BLASTN 2.2.16 [Mar-25-2007]


Reference: Altschul, Stephen F., Thomas L. Madden, Alejandro A. Schaffer,
Jinghui Zhang, Zheng Zhang, Webb Miller, and David J. Lipman (1997),
"Gapped BLAST and PSI-BLAST: a new generation of protein database search
programs",  Nucleic Acids Res. 25:3389-3402.

Query= SKboxes78-81-2_C21_sk16974_041.ab1
         (742 letters)

Database: pski015 blast database created by Steve 210708
           1 sequences; 10,138 total letters

Searching..................................................done



                                                                 Score    E
Sequences producing significant alignments:                      (bits) Value

|pski015| sequence of T-DNA used in generation of mutated popul...   188   1e-50

>|pski015| sequence of T-DNA used in generation of mutated population
          Length = 10138

 Score =  188 bits (95), Expect = 1e-50
 Identities = 101/103 (98%)
 Strand = Plus / Minus


Query: 63   attggtaattactctttcttttcctccatattgaccatcatactctttgctgatccatga 122
            ||||||||||||||||||||||||||||||||||||||||||||| ||||||||||||| 
Sbjct: 7805 attggtaattactctttcttttcctccatattgaccatcatactcattgctgatccatgt 7746


Query: 123  agatttcccggacatgaagccatttacaattgaatatatcctg 165
            |||||||||||||||||||||||||||||||||||||||||||
Sbjct: 7745 agatttcccggacatgaagccatttacaattgaatatatcctg 7703


Score = 99.6 bits (50), Expect = 8e-24
```

```
 Identities = 99/114 (86%), Gaps = 1/114 (0%)
 Strand = Plus / Minus


Query: 219  ttcccgaatgtgctccctaatggtgacgagggcataatcggacgctgacgccgcctcaac 278
            |||||| |||||||||| |||||||||||| |||||  |||||||| |||||||||| ||
Sbjct: 5999 ttcccgcatgtgctcccaaatggtgacgagcgcatagccggacgctaacgccgcctcgac 5940


Query: 279  aaccgccctcaccgcaaggaaggcgacgcgcagcctgatcacgcgggggcttct 332
            | ||||||||||||| ||||| || || |||||||| ||||||| || ||||||
Sbjct: 5939 atccgccctcaccgccaggaacgcaac-cgcagcctcatcacgccggcgcttct 5887
```

No. 9). Single imprecise left border sequence

```
BLASTN 2.2.16 [Mar-25-2007]


Reference: Altschul, Stephen F., Thomas L. Madden, Alejandro A. Schaffer,
Jinghui Zhang, Zheng Zhang, Webb Miller, and David J. Lipman (1997),
"Gapped BLAST and PSI-BLAST: a new generation of protein database search
programs",  Nucleic Acids Res. 25:3389-3402.

Query= SKboxes222-225-3_E20_sk31075_022.ab1
         (763 letters)

Database: pski015 blast database created by Steve 210708
           1 sequences; 10,138 total letters

Searching..................................................done



                                                                 Score    E
Sequences producing significant alignments:                      (bits) Value

|pski015| sequence of T-DNA used in generation of mutated popul...   444   e-127

>|pski015| sequence of T-DNA used in generation of mutated population
          Length = 10138

 Score =  444 bits (224), Expect = e-127
 Identities = 381/434 (87%)
 Strand = Plus / Minus


Query: 163  tttgcagccggtggagcttgcatgttggtttctacccagaaaagagccggtaaggaagat 222
            |||||| |||||||||||||||||||||||||||| |||||  |||||||| ||| ||||
Sbjct: 7689 tttgcacccggtggagcttgcatgttggtttctacgcagaactgagccggttaggcagat 7630


Query: 223  aatttgtattgtgaactgagccatgtacaccatccccccaacacggtgagcgaaggggca 282
            |||||  |||| |||||||||||||| |||| ||||||||||||||||||||| ||||||
Sbjct: 7629 aatttccattgagaactgagccatgtgcaccttccccccaacacggtgagcgacggggca 7570


Query: 283  acggagtgttctatatgggaattttaaacatcatccctcggatggagttgcgagagaggc 342
            |||||||| || | |||||| ||||||||||||||| |||||||| ||||||||||| ||
Sbjct: 7569 acggagtgatccacatgggacttttaaacatcatccgtcggatggcgttgcgagagaagc 7510


Query: 343  ggacgatccgtgatatcagccgtagcactgggcaggttcgtcccaagatcgcaaaggagt 402
             | |||||||||| ||||||||  |||| |||||||  ||   || |||||||||| | |
Sbjct: 7509 agtcgatccgtgagatcagccgacgcaccgggcaggcgcgcaacacgatcgcaaagtatt 7450


Query: 403  tgaacgtaggtacaatctagccgacgttcgcggaaccggaacgaccaagcaagctagctt 462
            |||||| |||||||||| ||||||||||| ||| ||||||||||||||||||||||||||
Sbjct: 7449 tgaacgcaggtacaatcgagccgacgttcacggtaccggaacgaccaagcaagctagctt 7390


Query: 463  gcggttcgccccggaacacgagcacggcacccgggaccactatgccaagagtgcccaagg 522
            ||  ||| |||| |||||||||||||||||||| ||||| |||||||||| |||||||||
Sbjct: 7389 gcaattcacccccgaacacgagcacggcacccgcgaccamtatgccaagaatgcccaagg 7330


Query: 523  taaaagtggcggggcccgctatgaagtccgtgaatgccccgagggccgaagtgagaggta 582
            ||||| | || || ||||| |||||||||||||||||||||| |||||||||||  || |
```

```
Sbjct: 7329 taaaaattgccggccccgccatgaagtccgtgaatgccccgacggccgaagtgaagggca 7270


Query: 583  ggccgccacccagg 596
             ||||||||||||||
Sbjct: 7269 ggccgccacccagg 7256



 Score =  147 bits (74), Expect = 4e-38
 Identities = 107/118 (90%)
 Strand = Plus / Minus


Query: 39   gcggacatcgacattttgaattgaaatacaattggtaattactctttcttttcctccat 98
             ||||||||| |||||||||||||||| | |||||||||||||||||||||||||||||||
Sbjct: 7836 gcggacatctacattttgaattgaaaaaaaattggtaattactctttcttttcctccat 7777


Query: 99   attgaccatcatactctctgctcctccacaaggatttcccggacatgaagccatttac 156
             ||||||||||||||||  ||||  ||||    ||||||||||||||||||||||||||
Sbjct: 7776 attgaccatcatactcattgctgatccatgtagatttcccggacatgaagccatttac 7719
```

No. 10). Two inserts nested inside T-DNA sequence

```
BLASTN 2.2.16 [Mar-25-2007]


Reference: Altschul, Stephen F., Thomas L. Madden, Alejandro A. Schaffer,
Jinghui Zhang, Zheng Zhang, Webb Miller, and David J. Lipman (1997),
"Gapped BLAST and PSI-BLAST: a new generation of protein database search
programs",  Nucleic Acids Res. 25:3389-3402.

Query= SKBOX12_C12_sk1170_087.ab1
         (830 letters)

Database: pski015 blast database created by Steve 210708
           1 sequences; 10,138 total letters

Searching..................................................done



                                                                 Score    E
Sequences producing significant alignments:                      (bits) Value

|pski015| sequence of T-DNA used in generation of mutated popul...   289   4e-81

>|pski015| sequence of T-DNA used in generation of mutated
           population
          Length = 10138

 Score =  289 bits (146), Expect = 4e-81
 Identities = 201/218 (92%), Gaps = 1/218 (0%)
 Strand = Plus / Plus


Query: 250 cagcttttgttccctttagggagggttaatttcaagcttgccctaatcatggccatagtt 309
           ||||||||||||||||||| ||||||||||| | |||||| | ||||||||| ||||| |
Sbjct: 1   cagcttttgttccctttagtgagggttaattccgagcttggcgtaatcatggtcatagct 60


Query: 310 gtttcctgcgcgaaattgttatccgctcacaattccacacacacatccgagccggaagca 369
           |||||||| | |||||||||||||||||||||||||||||| |||| |||||||||||||
Sbjct: 61  gtttcctgtgtgaaattgttatccgctcacaattccacaca-acatacgagccggaagca 119


Query: 370 tacagtgtaaagcctggggcgcctaatgagtgagctaactctcattaattgccttgcgct 429
           || |||||||||||||||| ||||||||||||||||||||| |||||||||| |||||||
Sbjct: 120 taaagtgtaaagcctggggtgcctaatgagtgagctaactcacattaattgcgttgcgct 179


Query: 430 cactgcccctttccagtcgggaaacctgtcgtgccag 467
           ||||||||  ||||||||||||||||||||||||||||
Sbjct: 180 cactgcccgctttccagtcgggaaacctgtcgtgccag 217
```

```
 Score =  103 bits (52), Expect = 5e-25
 Identities = 82/92 (89%)
 Strand = Plus / Minus

Query: 139  actctttttttttcctccatattgaccatcatactcattgttgattcccccacatttcccg 198
            ||||||| ||||||||||||||||||||||||||||||| |||| |    | ||||||||
Sbjct: 7795 actctttcttttcctccatattgaccatcatactcattgctgatccatgtagatttcccg 7736


Query: 199  gacatgaagccatttacaattgggtatatcct 230
            ||||||||||||||||||||||  ||||||||
Sbjct: 7735 gacatgaagccatttacaattgaatatatcct 7704
```
